# Social status, immune response and parasitism in males: a meta-analysis

**DOI:** 10.1098/rstb.2014.0109

**Published:** 2015-05-26

**Authors:** Bobby Habig, Elizabeth A. Archie

**Affiliations:** Department of Biological Sciences, University of Notre Dame, 100 Galvin Life Sciences Center, Notre Dame, IN 46556, USA

**Keywords:** social status, dominance rank, life-history trade-offs, immune function, parasitism

## Abstract

In male vertebrates, two conflicting paradigms—the energetic costs of high dominance rank and the chronic stress of low rank—have been proposed to explain patterns of immune function and parasitism. To date, neither paradigm has provided a complete explanation for status-related differences in male health. Here, we applied meta-analyses to test for correlations between male social status, immune responses and parasitism. We used an ecoimmunological framework, which proposes that males should re-allocate investment in different immune components depending on the costs of dominance or subordination. Spanning 297 analyses, from 77 studies on several vertebrate taxa, we found that most immune responses were similar between subordinate and dominant males, and neither dominant nor subordinate males consistently invested in predictable immune components. However, subordinate males displayed significantly lower delayed-type hypersensitivity and higher levels of some inflammatory cytokines than dominant males, while dominant males exhibited relatively lower immunoglobulin responses than subordinate males. Despite few differences in immunity, dominant males exhibited consistently higher parasitism than subordinate males, including protozoan blood parasites, ectoparasites and gastrointestinal helminths. We discuss our results in the context of the costs of dominance and subordination and advocate future work that measures both parasitism and immune responses in wild systems.

## 1. Introduction

Social hierarchies are a fundamental feature of many human and non-human animal societies [1]. In humans, socio-economic status (SES) has played a critical role in both historical and sociological contexts, manifesting itself in movements such as the French Revolution, and more recently, Occupy Wall Street and the emergence of the Tea Party [2–5]. An individual's position in a hierarchy can have striking effects on their health. Studies of humans have shown that individuals of lower SES suffer disproportionately from most documented diseases and exhibit higher rates of mortality relative to individuals of higher SES [6–8]. Social status is also often linked to health disparities in non-human animals, but the effects are mixed; sometimes low-status animals have worse health than high-status animals (e.g. [9–11]), and sometimes high-status animals exhibit worse health than low-status animals (e.g. [12–15]). These differences are puzzling.

In both humans and non-human animals, status-related differences in health are thought to be partly caused by status-related differences in immune function [16–18]. Here, we focus on these relationships in adult male vertebrates. Understanding the connections between social status and immune function in males is important because, in many species, high-status males engage in greater mating effort than low-status males, and these energetic costs of reproduction may result in trade-offs with survival-related tasks, including immune function [19–22]. Thus, discovering how immune responses vary with social status helps reveal how males allocate energy towards two major components of fitness—survival and reproductive effort.

To date, two disparate and somewhat contradictory paradigms have been proposed to explain hierarchy-related differences in male health and immune function. The first explanation, which is usually invoked to explain observations of low immune function in high-status males, is that the energetic costs of high social status, such as high reproductive effort and intense male–male competition, cause immunosuppression [23–28]. This trade-off may be partly mediated by testosterone, and sometimes glucocorticoids, which help direct energetic resources toward reproduction and away from tasks associated with long-term survival, such as immune function [13,23,24,26,27]. Hence, the greater intensity of effort displayed by high-status males via reproductive effort, maintenance of rank, and the subsequent differences in social organization, diet and foraging behaviours may mean that high and low-status males effectively occupy different socio-ecological niches, leading to differences in immune function or parasitism [29–31]. In support, some studies have shown that investment in reproduction and/or elevated testosterone is associated with decreased immunity [27,32]. Similarly, other studies have shown that investments in reproductive effort via testosterone production and/or body ornamentation positively correlate with parasite load [14,28,33–37].

Conversely, a second explanation, usually invoked to explain low immune function in low-status males, is that status-related differences in immune function are caused by differences in exposure to chronic stress [16,38–40]. Low-status males may be more likely to experience unpredictable events, or are less able to cope with these events, leading to chronically elevated glucocorticoid levels and ultimately immunosuppression [16,39,41]. In support, there is strong evidence that the cumulative physiological burdens associated with chronic stress tend to depress immune function [40,42] especially in low-status individuals [9,43–47]. Furthermore, in many societies, low social status in males is generally associated with poor health and elevated disease risk [16,18,41,48]. Studies have also shown that chronic psychological stress tends to suppresses cell-mediated (Th-1) defences [49] and enhance pro-inflammatory responses [50], and that Th-2 cells generally stimulate the production of pro-inflammatory IL-6 [51], which transiently proliferates following exposure to physical and psychological stressors [52,53].

Despite decades of research on both of these paradigms, neither has provided a complete explanation for status-related differences in male health and immunity [40,54,55]. One challenge is that these two paradigms are rarely examined simultaneously in the same species or population. This is important because, while both explanations have been partly successful in explaining some aspects of status-related differences in immunity, they explain two seemingly incongruent phenomena: immunosuppression in high-status males (e.g. [12–15]) versus immunosuppression in low-status males (e.g. [9–11]). A second challenge is that studies testing these ideas have tended to oversimplify the vertebrate immune system, often relying on only one or a few assays to evaluate male immune responses in any given species or population [18,48,56,57]. However, the vertebrate immune system is multifaceted, with several semi-independent modes of response that can be upregulated or downregulated depending on the diseases or injuries organisms face and their energetic limitations [58,59]. A third challenge is that the nature of dominance hierarchies can vary within and between species, and the criteria used to assign rank can vary between populations and studies [16]. For instance, in humans, high and low status are often distinguished by measures of SES, occupation and educational levels [60]. In non-human animals, hierarchies can be delineated by physiological, ecological or behavioural parameters and can vary in their strength, linearity and stability [16]. In this paper, we considered dominance ranks to represent any asymmetrical relationship in which one or more individuals consistently outcompeted others in dyadic agonistic interactions [61].

We attempted to address these challenges by drawing on ideas from ecoimmunology that take a pan-immune system approach to understanding adaptive variation in immune response. Under this perspective, organisms are not expected to experience broad immunosuppression in the face of energetic or hormonal challenges; rather they should reallocate their investment in different types of immune defence depending on their energetic and disease-related costs [31,54,57,58]. To date, two such hypotheses have been proposed that make specific predictions about how males should allocate investment in immune defence as a function of reproductive effort or stress.

### (a) The trade-offs model: hypothesis and predictions

**Figure 1.**
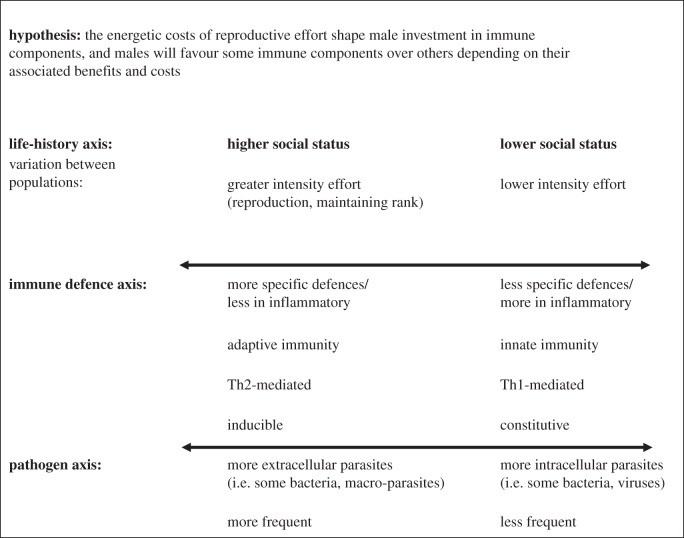
The trade-offs model, modified from Lee [31].

The first hypothesis (figure 1), adapted from a framework developed by Lee [31], proposes that the energetic costs of reproductive effort shape male investment in immune components, and that males will favour some immune components over others depending on their associated benefits and costs [57,58]. This hypothesis categorizes immune defences based on multiple dimensions (table 1)—i.e. inducible versus constitutive defences, specific versus non-specific defences [57,58] and Th-1 mediated versus Th-2 mediated defences [31]. Dominant males, especially those that engage in high reproductive effort, are predicted to favour less energetically costly immune defences (i.e. inducible and specific defences) and anti-inflammatory defences (i.e. Th-2 mediated), while subordinate males will favour non-specific, constitutive and inflammatory defences (i.e. Th-1 mediated) [31,57]. In terms of parasitism, this model predicts that, due to differential exposure to parasites, high-status males will be at greater risk for extracellular parasites than low-status males, while low-status males will be at greater risk for intracellular parasites than high-status males [31].

**Table 1.** Immune system components used for assessing the effects of social status on immune function.

immune component	description	examples	references
innate (non-specific)	host defences that exist before antigen exposure; generally confers non-specific and constitutive immune defences although inducible and specific properties are critical in certain innate defences. Three main defences are: phagocytosis, inflammation and the complement cascade	macrophages, neutrophils, basophils, eosinophils, natural killer (NK) cells and antimicrobial peptides/proteins (complement, defensins, c-reactive proteins)	[62,63]
adaptive (specific)	host defences that are mediated by antigen exposure and the activation of B and T cells. Adaptive components of the immune system exhibit highly diverse specificity to pathogens, retention of immunological memory and non-self-recognition	B lymphocytes, T lymphocytes, T helper cells, T cytotoxic cells, antibodies	[62,64]
constitutive	components of either the innate or adaptive arm of immunity that are expressed at all times; a non-induced form of immune function; confers a first line of defence against pathogens prior to pathogen-specific antigen exposure	examples of constitutive innate components: marcrophages, heterophils, granulocytes, NK cells and various antimicrobial peptides/proteins examples of constitutive adaptive components: naturally circulating antibodies (e.g. IgM)	[31,57,58,65,66]
inducible	components of either the innate or adaptive arm of immunity that are expressed following challenge by a pathogen; innate components induce inflammatory responses and increase rates of immune responses; adaptive components induce immunological memory, opsonization of pathogens and cell-mediated responses	examples of inducible innate components: production of reactive oxygen species (ROS) and cytokines by macrophages and granulocytes examples of inducible adaptive components: B lymphocytes, T helper cells, antibodies	[31,57,58]
Th-1 mediated	subset of adaptive immunity; secretes a unique profile of cytokines; Th-1 cells provide cellular immunity against intracellular bacteria, protozoa, fungi and viruses, help to eradicate cancer cells and stimulate delayed-type hypersensitivity (DTH) inflammatory reactions; important for macrophage and cytoxic T-cell activation	acute phase responses; cytokines including IFN-*γ*, TNF-*α*, TNF-*β*, TGF-*β*	[51,67–70]
Th-2 mediated	subset of adaptive immunity; secretes a unique profile of cytokines; Th-2 cells provide humoral immunity against helminths and other extracellular pathogens; stimulates B cell, eosinophil and mast cell production and is subsequently important in the upregulation of antibody formation; induces B-cell class switching	antibody production; cytokines including IL-4, IL-5, IL-6, IL-10 and IL-13	[51,67–70]

### (b) The stress–response model: hypothesis and predictions

**Figure 2.**
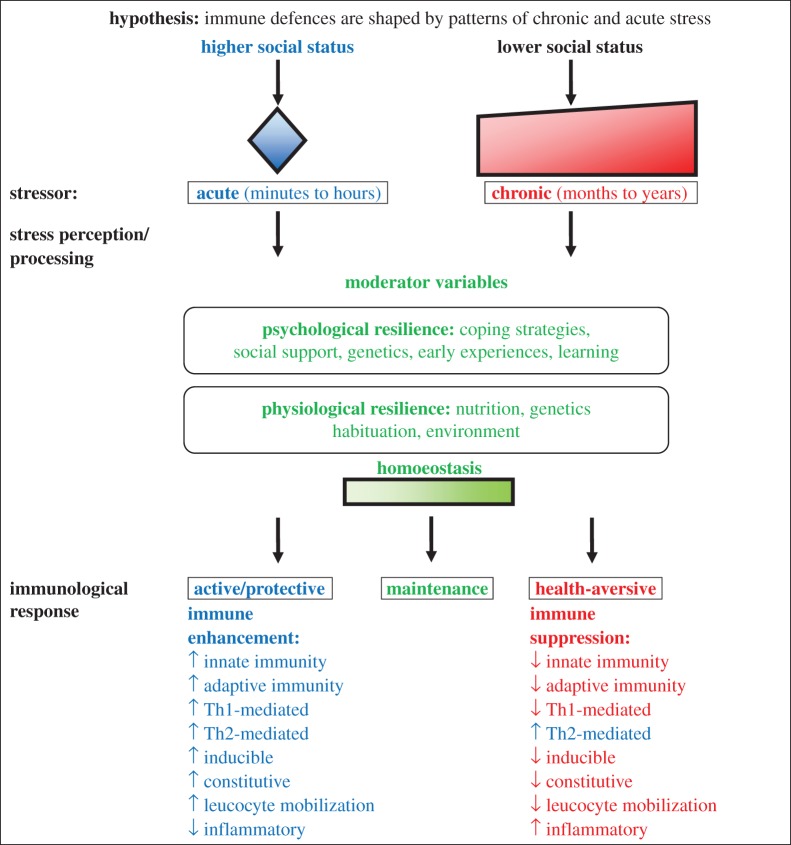
The stress–response model, modified from Dhabhar [40]. In this model, blue represents immune enhancement, green represents homoeostasis and red represents immune suppression/dysregulation.

A second hypothesis (figure 2), based on a framework developed by Dhabhar [40], proposes that immune defences will be shaped by patterns of chronic and acute stress. In this hypothesis, stressors serve antagonistic functions—in some cases facilitating immunity and preparing the body for challenges to the immune system, and in other circumstances dysregulating immune responses, causing sickness and disease [40]. Among stable societies, individuals exposed to short-term, *acute* stressors, generally high-ranking individuals [16,41,71], are predicted to have enhanced innate (non-specific), constitutive, adaptive (specific) and induced immune responses. Conversely, among stable societies, individuals exposed to *chronic* stress, generally low-ranking individuals [16,41,71], are expected to exhibit mostly immunosuppressive responses. Furthermore, chronic stress is predicted to enhance type-2 mediated immunity and to suppress type-1 mediated responses [72].

We tested predictions of these hypotheses in a meta-analytical framework to gain a more complete understanding of how social status affects immune responses and measures of parasitism in male vertebrates.

## 2. Material and methods

### (a) Identification of studies and inclusion criteria

To identify published studies on the association between male social status and immune responses, we conducted an extensive electronic search in Web of Science. The literature search took place between January and February 2014, and the years covered in the search spanned 1900–2014. We searched for all possible pair-wise combinations of two search terms, one each from either (i) social hierarchy, social dominan* and social status or (ii) disease*, parasit*, immune function and health. In addition, we searched the bibliographies of highly cited and/or recent review articles on social status and immunity to supplement the Web of Science electronic search [16,18,41,48]. We accepted both experimental and observational studies, and we accepted studies published in all languages.

To be included in the meta-analysis, the study species had to be a member of the vertebrate sub-phylum, including both captive and free-living populations. In addition, immune responses of dominant males had to be directly compared to immune responses of subordinate males. While we conceptually defined dominance relationships based on asymmetrical, competitive interactions [61], operationally, these included diverse measures of individual behaviour, morphology, physiology and condition (electronic supplementary material, table S1). Studies that compared dominants or subordinates with ‘controls’ (e.g. socially isolated animals) were excluded. We also excluded analyses that included juveniles, mixed sexes or castrated males.

### (b) Data extraction

We extracted several types of data from each study: (i) citation information, including the journal and authors; (ii) the species involved; (iii) the study setting as captive (laboratory or zoo animals), wild (non-provisioned, free-ranging animals) or semi-natural (provisioned animals or wild animals kept in captivity only during immune tests); (iv) the method of measuring dominance rank (electronic supplementary material, table S1); (v) the types of immunological (electronic supplementary material, table S2) or parasitological (electronic supplementary material, table S3) measures used to test status-related differences in health; (vi) when relevant, the component of immune defence these measures reflected (table 1); and (vii) the effect sizes, measures of dispersion, sample sizes and *p*-values for each test of immune response included in the study. Parasitological measures included estimates of parasite prevalence, parasite species richness and infection intensity. For studies that represented their results graphically, but did not report exact numerical results, we used Web Plot Digitizer v. 3.3 [73] to extract means and standard errors or means and standard deviations from relevant figures, and then converted them to standardized mean differences. All data were compiled by one author and checked by a second.

### (c) Statistical analyses

To quantify the effects of social status on immune response, we used a meta-analytic approach. We designated dominant males as the control group and assigned subordinates to the treatment group. An effect size calculator [74] was used to convert means and standard errors, means and standard deviations, *t*-tests and *p*-values, as well as other statistical measures, to a standardized mean difference, Cohen's *d*. We then checked these measures using the compute effect sizes [*compute.es*] package [75] in R [76]. Cohen's *d* was used as a measure of effect size and to summarize differences between dominants and subordinates. We determined significance by calculating the 95% confidence intervals (CI) surrounding *d*, which has an unbounded range [77]. Significantly positive values represented studies in which subordinate males exhibited higher immune responses than dominant males. Significantly negative values represented studies in which dominant males yielded higher immune responses than subordinate males. In the case of parasitism, significantly positive values reflected lower parasitism in subordinate relative to dominant males and vice versa. We rejected the null hypothesis of no effect when effect sizes differed significantly from zero. For studies that included multiple time points for a given test of immune response, we chose data from the median time point to calculate the effect size. Owing to the relatively low taxonomic diversity, we did not account for phylogeny in our meta-analyses.

Before addressing our specific hypotheses, we first tested for significant differences between dominants and subordinates for each individual test of immune response (electronic supplementary material, table S2) or measure of parasitism (electronic supplementary material, table S3). We also tested one measure of physical condition, haematocrit, which indicates anaemia [62], and may reflect costs of parasites that consume blood (electronic supplementary material, table S3). We only conducted meta-analyses when three or more studies were available for a given test of immune response or parasitism. We performed tests of significance using the R *metafor* package [78], and we generated random effects models using the rma.mv function. As some studies performed the same test on multiple, independent populations, we applied the restricted maximum-likelihood method and performed a multivariate meta-analysis to account for correlation between outcomes. In such cases, we modelled study identity as a random effect. Furthermore, as the literature review yielded studies of multiple species, we categorized species into seven vertebrate classes/orders (Actinopterygii, Artiodactyla, Aves, Carnivora, Primates, Rodentia and Squamata) and treated taxa as a moderator variable. When taxa had no significant effect on the model outcome, we excluded this moderator from the final analysis.

We next tested the specific predictions of the trade-offs and the stress–response hypotheses (figures 1 and 2). We did so by combining studies that reflected similar immune components (electronic supplementary material, table S4). Note that most tests of immune response were included in multiple tests of immune components. For instance, baseline immunoglobulin levels (row 1 in electronic supplementary material, table S2) measure adaptive, constitutive, and Th-2 mediated immunity [79,80] and were included in tests of all of these immune components. For Th-1- and Th-2-mediated immunity, we also completed three sub-analyses (electronic supplementary material, table S5): (i) one that separately assessed all available Th-1 cytokines (IFN-1, IFN-*γ* and TNF-*α*); (ii) one that separately assessed all available Th-2 cytokines (IL-4, IL-6 and IL-10); and (iii) one that assessed all available pro-inflammatory cytokines (IFN-1, IFN-*γ*, TNF-*α* and IL-6). To test the links between social status and individual disease risk, we conducted a meta-analysis of the relationships between dominance rank and measures of parasitism (electronic supplementary material, tables S3 and S6). Finally, we conducted supplementary meta-analyses to assess patterns for the taxa that contributed the largest number of studies to our sample: (i) Rodentia, (ii) Primates and (iii) Aves. As before, we conducted meta-analyses for sample sizes of three or more. We applied random effects models and tests of significance using the *rma.mv* function in the *metafor* package [78]. As some tests included multiple outcomes from the same study (i.e. two different tests of immune response on the same population), we modelled study identity as a random effect. We treated taxa and test of immune response as moderator variables, but these factors were excluded from the final models if they had no significant effect on model outcome. Finally, we assessed publication bias visually via funnel plot analyses and quantitatively using Egger's tests [81].

## 3. Results

### (a) Characteristics of the studies used in meta-analyses

Our literature search yielded 77 studies that met the criteria for our meta-analyses, including 297 distinct analyses of male immune responses or parasitism. These studies were found in 44 distinct scientific journals (electronic supplementary material, figure S1). We excluded 35 studies because they did not report effects sizes and/or sample sizes. Among the 77 accepted studies, we identified 34 different tests of immune response (electronic supplementary material, figure S2) and three categories of parasitism (ectoparasites, gastrointestinal parasites and blood parasites). Five orders and two classes of vertebrates were represented across analyses (electronic supplementary material, figure S3). The most common taxonomic group was rodents (Rodentia; 59%; *n* = 45 studies; electronic supplementary material, figures S4 and S5), followed by primates (Primates; 18%; *n* = 14; electronic supplementary material, figure S6) and birds (Aves; 13%; *n* = 10). The remaining taxa included ray-finned fishes (Actinopterygii; *n* = 4), even-toed ungulates (Artiodactyla; *n* = 2), carnivores (Carnivora; *n* = 1) and lizards (Squamata; *n* = 1). In terms of study setting, 81% of studies (*n* = 62) were conducted in captivity, 17% occurred in the wild (*n* = 13), and 3% took place in a semi-natural environment (*n* = 2). Overall, these studies used 16 different methods to measure male dominance status, sometimes using multiple measures in the same study (electronic supplementary material, table S1).

### (b) Neither dominant nor subordinate males consistently demonstrated reduced immunity

**Figure 3.**
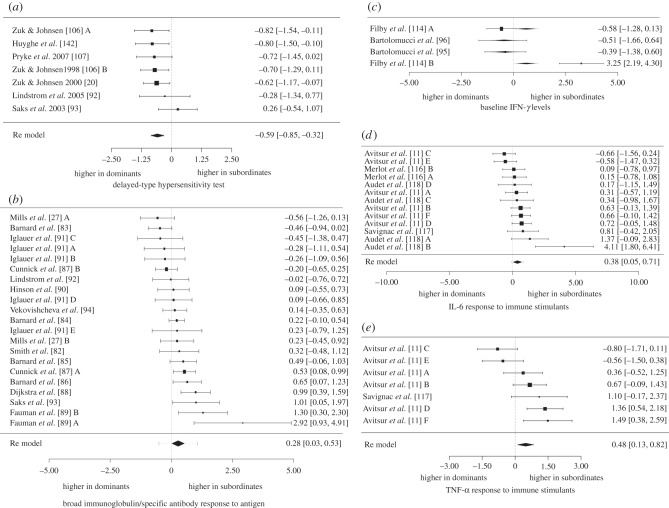
Forest plots showing effect sizes for the five tests of immune response that demonstrated a significant relationship with social status in table 2. Positive values indicate higher responses in subordinates; negative values indicate higher responses in dominants. Plots include the effect sizes for (*a*) DTH responses to immune stimulants, (*b*) immunoglobulin responses to antigens, (*c*) baseline IFN-*γ* levels, (*d*) IL-6 response to immune stimulants and (*e*) TNF-*α* response to immune stimulants. Values in brackets represent the 95% CI lower limit and the 95% CI upper limit; the value outside brackets represents the effect size (*d*) of each study. Letters following an author's name represent studies of the same test on multiple, independent populations. In (*c*), which shows baseline IFN-*γ* levels, grey diamonds represent a fitted value for each study that incorporates taxa as a moderator.

We found little evidence that either dominant or subordinate males consistently demonstrated low immune responses. For instance, among the 31 tests of immune response with three or more analyses, we only observed one test where subordinate males exhibited significantly lower responses than dominant males (table 2). Moreover, when we restricted our analysis to the eight tests of immune response with a sample size of 10 or more analyses, there were no tests where subordinate males displayed significantly lower immune responses than dominant males. The only test where subordinate males displayed significantly lower immune responses than dominant males was delayed-type hypersensitivity (DTH), an inducible measure of adaptive, Th-1 mediated responses [79,80] (*d* = –0.586; *p* < 0.0001; *n* = 7; figure 3*a*). However, five of the seven DTH analyses were performed on birds; hence, this pattern may be taxonomically biased.

**Table 2.** Summary of meta-analyses for individual tests of immunity.

			random effects model					
test of immune function	sample size (analyses)	Cohen's *d*	95% CI lower limit	95% CI upper limit	*z*-value	*p*-value	higher in dominant or subordinate	citations
baseline immunoglobulin levels	3	−0.256	−0.673	0.161	−1.203	0.229	neither	[27,82]
broad immunoglobulin response/production of a specific antibody in response to antigen	21	0.277	0.026	0.527	2.165	0.03	subordinate	[27,82–94]
*in vitro* lymphocyte proliferation to mitogens (combined)	26	−0.297	−1.045	0.452	−0.777	0.437	neither	[95–105]
*in vitro* lymphocyte proliferation to concanavalin A (Con A)	16	−0.042	−0.707	0.622	−0.125	0.901	neither	[95–98,100–105]
*in vitro* lymphocyte proliferation to phytohemagglutinin (PHA)	6	−0.381	−1.295	0.534	−0.816	0.416	neither	[97,100–102]
skin swelling test after initial exposure (delayed-type hypersensitivity; DTH)	7	−0.586	−0.849	−0.093	−0.324	<0.0001	dominant	[20,92,93,106,107]
haemagglutination assay^a^	2	n.a.	n.a.	n.a.	n.a.	n.a.	n.a.	[108,109]
haemolysis/haemolytic complement assay	3	−0.184	−0.704	0.337	−0.692	0.489	neither	[108,110]
macrophage phagocytic ability	3	−0.184	−0.721	0.352	−0.673	0.501	neither	[10,111]
natural killer (NK) cell cytotoxicity	9	–0.213	−0.771	0.344	−0.750	0.453	neither	[10,100,104,105,112,113]
baseline IFN-*γ* levels^b^	4	0.610	0.022	1.197	2.034	0.042	subordinate	[95,96,114]
baseline TNF-*α* levels	3	−0.298	−0.796	0.200	−1.172	0.241	neither	[114,115]
IFN-*γ* response to immune stimulants	6	0.027	−0.385	0.439	0.128	0.898	neither	[95,96,116,117]
IL-1/IL-1*β* response to immune stimulants	9	0.317	−0.804	1.437	0.554	0.580	neither	[117,118]
IL-2 response to immune stimulants	7	−0.468	−1.266	0.330	−1.150	0.250	neither	[95,96,119]
IL-4 response to immune stimulants	3	−0.400	−1.005	0.205	−1.295	0.196	neither	[95,96]
IL-6 response to immune stimulants	13	0.387	0.049	0.726	2.244	0.025	subordinate	[11,116–118]
IL-10 response to immune stimulants	8	−0.136	−0.679	0.408	−0.490	0.624	neither	[95,96,117,118]
TNF-*α* response to immune stimulants	7	0.476	0.128	0.824	2.679	0.007	subordinate	[11,117]
experimental infection with a pathogen or parasite	33	0.363	−0.060	0.787	1.685	0.092	neither	[10,33,84,90,91,120–127]
sickness behaviours and/or fever in response to an antigen^a^	2	n.a.	n.a.	n.a.	n.a.	n.a.	n.a.	[128]
skin swelling response to injection with mitogens/antigens	10	−0.041	−0.498	0.416	−0.176	0.860	neither	[129]
cell counts (B cells)	3	−0.076	−0.947	0.795	−0.170	0.865	neither	[103,104,121]
cell counts (cytotoxic T cells)	5	−0.051	−0.393	0.292	−0.290	0.772	neither	[97,103,104,130]
cell counts (granulocytes)	5	0.432	−0.216	1.080	1.306	0.192	neither	[103,104,130,131]
cell counts (helper T cells)	5	−0.287	−0.681	0.107	−1.427	0.154	neither	[97,103,104,130]
cell counts (leucocytes)	5	0.047	−0.361	0.455	0.225	0.822	neither	[93,103,105,130]
cell counts (lymphocytes)	15	0.133	−0.220	0.485	0.738	0.461	neither	[88,93,97,103,104,106,113,130–135]
cell counts (neutrophils)	3	−0.270	−1.399	0.859	−0.468	0.640	neither	[93,106,134]
neutrophil to lymphocyte ratio	3	−0.234	−1.356	0.888	−0.409	0.682	neither	[93,134,136]
adrenal mass	7	0.853	−0.145	1.851	1.676	0.094	neither	[90,136–139]
spleen mass^b^	16	−1.722	−3.819	0.375	−1.609	0.108	neither	[11,90,94,131,136,137,139–141]
thymus mass	9	−0.443	−1.424	0.538	−0.885	0.376	neither	[90,94,102,136,137,139,141]
size of spleen^a^	1	n.a.	n.a.	n.a.	n.a.	n.a.	n.a.	[131]

^a^Meta-analyses were only performed for sample sizes of three or more.

^b^The moderator variable (taxa) significantly explains between-study heterogeneity for baseline IFN-*γ* levels and spleen mass.

Dominant males were also not consistently immunosuppressed relative to subordinate males. Dominant males exhibited significantly lower immune responses than subordinate males in four of 31 tests of immune response (table 2). When we restricted the dataset to tests with 10 or more analyses, dominant males exhibited lower immune responses than subordinate males for two of eight tests (table 2). In particular, dominant males exhibited significantly lower immunoglobulin responses to antigen challenge than subordinate males, a test that reflects one aspect of adaptive, inducible and Th-2 mediated immunity [79,80] (*d* = 0.277; *p* = 0.03; *n* = 21; figure 3*b*). For the three remaining significant tests, all were measures of pro-inflammatory cytokines. Specifically, subordinate males exhibited relatively greater baseline levels of IFN-*γ* (*d* = 0.610, *p* = 0.042; *n* = 4; figure 3*c*) and higher IL-6 and TNF-*α* responses to immune stimulants than dominant males (IL-6: *d* = 0.387; *p* = 0.025, *n* = 13; figure 3*d*; TNF-*α*: *d* = 0.476; *p* = 0.007; *n* = 7; figure 3*e*). These results indicate that subordinate males may exhibit dysregulated inflammatory responses relative to dominant males, and probably should not be taken as evidence for stronger immune function in subordinate than dominant males. Lastly, when we combined all individual tests of immunity into a single meta-analysis, there was no significant difference between dominant and subordinate males (*d* = 0.082; *n* = 282; *p* = 0.36).

### (c) Neither the trade-offs hypothesis nor the stress–response hypotheses were well supported

Ecoimmunologists predict that organisms should invest in different immune components depending on their energetic and disease-related costs [31,54,57,58]. However, meta-analyses of the six immune components revealed little support for the idea that dominant or subordinate males consistently invest in certain immune components (table 3; see electronic supplementary material, table S3 for tests and sample sizes).

**Table 3.** Summary of meta-analyses for tests of immune system components.

				random effects model				
test of immune function	sample size (analyses)	Cohen's *d*	Egger's test (*p*-value)	95% CI lower limit	95% CI upper limit	*z*-value	*p*-value	higher in dominant or subordinate
tests of adaptive immunity	57	−0.080	0.063	−0.390	0.230	−0.506	0.613	neither
tests of innate immunity	17	−0.062	0.425	−0.757	0.634	−0.173	0.863	neither
tests of induced immunity	154	0.083	0.641	−0.152	0.317	0.691	0.490	neither
tests of constitutive immunity	25	0.046	0.386	−0.462	0.554	0.178	0.859	neither
tests of Th-1 mediated immunity	29	−0.145	0.096	−0.427	0.136	−1.0122	0.312	neither
tests of Th-2 mediated immunity	55	0.282	0.068	−0.052	0.616	1.653	0.098	neither
Th-1 cytokines	22	0.062	0.348	−0.216	0.339	0.437	0.662	neither
Th-2 cytokines	29	0.208	0.063	−0.101	0.516	1.320	0.187	neither
inflammatory cytokines (IFN-1, IFN-*γ*, IL-6, TNF-*α*)	37	0.190	0.018	−0.082	0.462	1.372	0.170	neither

The trade-offs hypothesis predicted that dominant males would invest in adaptive, inducible and Th-2 mediated responses, while subordinate males would invest in innate, constitutive and Th-1 mediated responses [31]. However, we found no significant differences in the immune responses of dominant and subordinate males for any of the six immune components (table 3). These models were not significantly improved by including either taxa or test of immune response as moderator variables. Furthermore, the patterns were largely the same when we repeated these immune component meta-analyses for the three most frequent taxa in our dataset: rodents, primates and birds (electronic supplementary material, table S7). The only exception was that, in birds, dominant males had significantly higher adaptive immune responses than subordinate males (*d* = −0.689, *p* < 0.0001, *n* = 6). However, five of these studies (83%) used DTH as a measure of adaptive immune response; hence, it is unclear whether this pattern reflects the use of other tests of adaptive immunity in birds.

The stress–response hypothesis predicted that dominant males would invest in adaptive, innate, inducible, constitutive and Th-1 immune responses, while subordinate males would exhibit higher inflammatory responses compared to dominant males [40]. Support for this hypothesis was limited. There was little evidence that dominant males exhibited greater responses than subordinate males for any of the immune components, with the possible exception of adaptive immunity in birds (electronic supplementary material, table S7). Furthermore, while we observed some evidence that subordinate males exhibited elevated inflammation in individual tests of immune response (table 2), this analysis was not significant when we analysed all inflammatory cytokines together (table 3). Moreover, there was evidence for publication bias in combined tests of inflammatory cytokines (Egger's test: *p* = 0.018; electronic supplementary material, figure S7). Specifically, there were fewer analyses than expected with high inflammatory markers in dominants and small sample sizes. Finally, the stress–response hypothesis predicted that dominant and subordinate males make similar investments in Th-2 mediated responses. In support, we found no significant differences between dominant and subordinate males in the strength of Th-2 responses (table 3; electronic supplementary material, table S7). Funnel plots for immune components are shown in the electronic supplementary material, figures S8–S15.

### (d) Dominant males almost always had higher parasitism than subordinate males

**Figure 4.**
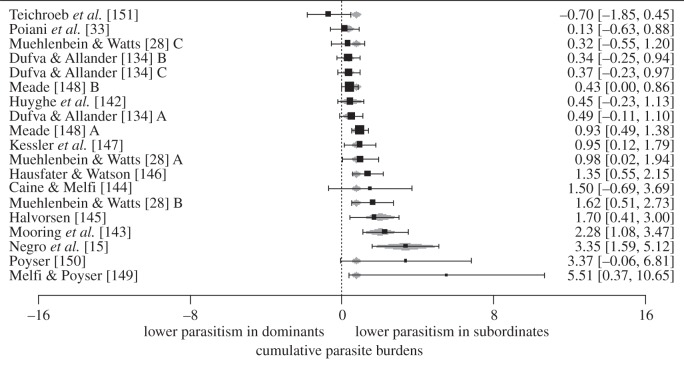
A forest plot showing the effect sizes of all studies that tested the effects of social status on patterns of parasitism. Positive values indicate lower parasitism in subordinates; negative values indicate higher parasitism in dominants. The values in brackets represent the 95% CI lower limit and the 95% CI upper limit; the value outside brackets represents the effect size (*d*) of each study. Letters following an author's name represent studies of the same test on multiple, independent populations. Grey diamonds represent a fitted value for each study that incorporates taxa as a moderator.

Despite few differences in immune responses, we found that dominant males experienced greater parasitism than subordinate males (table 4; figure 4). Specifically, compared with subordinate males, dominant males were significantly more likely to experience greater measures of ectoparasites (*d* = 2.275; *p* = 0.0002; *n* = 3), blood parasites (*d* = 0.401; *p* = 0.024; *n* = 3) and gastrointesinal parasites (*d* = 1.201; *p* = 0.0017; *n* = 13). When we restricted the dataset to gastrointesinal helminths, dominant males were significantly more parasitized than subordinate males (*d* = 1.445; *p* < 0.001; *n* = 10). When all individual tests of parasitism were combined, dominant males were also significantly more parasitized than subordinate males (*d* = 2.015; *p* < 0.0001; *n* = 19; figure 4). There was a non-significant trend for publication bias for this test (*p* = 0.058; electronic supplementary material, figure S16), and the taxonomic group of the subjects explained significant between-study heterogeneity and was included as a moderator variable (*p* = 0.0002; table 4). Interestingly, the patterns of parasitism we observed were also consistent with measures of haematocrit. Animals may exhibit low haematrocrit when they are heavily infected with parasites that consume blood, including many ectoparasites and helminths [152–154]. In support, we found that dominant males had significantly lower haematocrit levels than subordinate males (*d* = 0.638; *p* = 0.0016; *n* = 4), perhaps reflecting higher parasitism in dominants.

**Table 4.** Summary of meta-analyses for measures of parasitism, tests of condition and cumulative parasitism.

			random effects model					
measure of parasitism**^a^** or condition	sample size (analyses)	standard difference in means	95% CI lower limit	95% CI upper limit	*z*-value	*p*-value	lower in dominant or subordinate	citations
blood parasites	3	0.401	0.053	0.749	2.257	0.024	subordinate	[134]
ectoparasites^b^	3	2.275	1.085	3.465	3.746	0.0002	subordinate	[33,143,144]
gastrointestinal parasites	13	1.201	0.549	1.853	3.611	0.0017	subordinate	[15,28,145–151]
gastrointestinal parasites (helminths only)^c^	10	1.445	0.879	2.012	5.038	<0.0001	subordinate	[15,28,145–150]
all parasite types^d,e^	19	2.015	1.136	2.892	4.495	<0.0001	subordinate	
*tests of condition:* haematocrit^f^	4	0.638	0.241	1.035	3.147	0.0016	subordinate	[114,131,141]

^a^Measures of parasitism included estimates of parasite infection prevalence, parasite species richness and parasite infection intensity.

^b^Taxa significantly explains between-study heterogeneity for tests of ectoparasites.

^c^Gastrointestinal helminths were assessed as a sub-category of all GI parasites.

^d^Taxa significantly explains between-study heterogeneity for tests of cumulative parasites.

^e^Egger's test: *p* = 0.058.

^f^Taxa significantly explains between-study heterogeneity for tests of haematocrit.

The observation that dominant males experienced higher parasitism than subordinate males, and that dominant and subordinate males demonstrated few differences in immunity, is intriguing. One reason for this pattern may be that the majority of studies measuring immune responses were performed on captive populations (89%; 59 of 66 studies) while most studies of parasitism were performed on either semi-natural or wild populations (79%; *n* = 11 of 14 studies). However, when we analysed the seven wild/semi-natural studies of immune response, we found no significant differences between dominant and subordinate males (*d* = 0.013, *n* = 16, *p* = 0.96). Moreover, there was some evidence that dominant males experience higher disease risk than subordinate males, even in captivity. Specifically, in individual tests of immune response, we found that dominant males produced significantly fewer antibodies to antigen challenge (figure 3*b*; table 2; *d* = 0.277; *n* = 21; *p* = 0.03; 100% of these studies were captive), and we also detected a non-significant trend for dominant males to be more susceptible to experimental infections with parasites (table 2; *d* = 0.363; *n* = 33, *p* = 0.092; 97% of these analyses were captive). It is also possible that taxonomic biases explain the differences between parasitism and immune response, as most immune tests were performed on rodents (68%; 45 of 66 studies), while most tests of parasitism were performed on primates (57%; 8 of 14 studies). However, dominant males still had higher parasitism than subordinate males, even when we excluded primates from the dataset (*d* = 0.822, *n* = 8, *p* < 0.0001).

## 4. Discussion

Our meta-analytic review yielded two primary results: (i) neither dominant nor subordinate males consistently demonstrated reduced immunity, and (ii) dominant males almost always experienced greater parasitism than subordinate males. Specifically, immune responses were most often similar in dominant and subordinate males, but subordinate males sometimes exhibited elevated markers of inflammation and significantly lower DTH than dominant males. By contrast, dominant males exhibited significantly lower immunoglobulin responses and greater parasitism than subordinate males. Recently, ecoimmunologists have proposed that individuals should differentially invest in different immune components (e.g. adaptive versus innate immunity; inducible versus constitutive immunity) depending on the disease risks and energetic costs they face [31,54,57,58]. However, we found little evidence that, across species, either high- or low-ranking males consistently invest in a predictable set of immune components. Here we discuss the implications of our results, including prospects for the trade-offs and stress–response models, possible reasons why we observed differences in parasitism but not immunity, and useful directions for future research.

### (a) The trade-offs and the stress–response models

Our meta-analyses revealed little support for either the trade-offs or the stress–response models. This lack of support is probably due to several factors, but one primary reason was that many of the tests of immune response we reviewed were conducted in captive settings, which may affect the costs of male rank. Specifically, our analyses rested on two assumptions: (i) that dominant males experience higher energetic costs than subordinates, either as a result of reproductive effort or agonistic conflict and (ii) that subordinate males, rather than dominant males, experience chronic stress. Many of these assumptions are well supported for the species we included in our meta-analyses. Indeed, the energetic costs of male reproductive effort have been documented in numerous taxa, including primates [28,155,156], rodents [140], birds [157,158] and ungulates [159–161]. For example, in wild chimpanzees, dominant males invest a considerable proportion of time attaining and maintaining dominance rank, and this effort appears to be traded off with helminth parasite richness [28]. Likewise, the costs of chronic stress have also been documented across numerous taxa, including primates [16,41,43,132,162], rodents [11,47], birds [106] and fishes [114]. However, the majority of tests of immune response available for meta-analyses were conducted on males housed in captivity (89% of studies). These males probably did not experience natural opportunities for male–male competition and reproduction. In addition, in captivity, subordinate males may be less able to escape aggressive targeting by dominants, potentially exacerbating chronic stress in captive versus wild settings [163–165]. Hence, the males in our meta-analyses may not have experienced the same costs of high and low rank as they would in a natural population, limiting our abilities to fully test the trade-offs or the stress–response models.

In addition to the effects of study setting, the costs of social status probably also vary across species and social systems. For instance in humans, SES appears to be a robust predictor of health and disease risk, but evidence from non-human animals is more equivocal [16]. Some of these differences might be caused by a lack of social mobility between humans from generation to generation as compared to animals, but the underlying causes remain an open question. Variation in social organization and the stress associated with low versus high social status in different societies is also likely to be an important determinant of status-related differences in health [163] (but see [166]). Furthermore, the type of mating system probably also plays an important role in status-related variation in immune responses [167]. Finally, dominance hierarchies vary across species in their strength, stability and linearity, and these differences probably also have important consequences for male immune function [16,41]. Thus, both the taxa and the dominance structures in our meta-analyses may have been too diverse for either the trade-offs or the stress–response models to be broadly predictive. However, even when we restricted our analyses to each of the three most common taxonomic groups (i.e. rodents, primates or birds), the trade-offs and the stress–response models were still not well supported. This finding suggests that male investment in different immune components may be relatively species specific, making it challenging to develop hypotheses that accurately predict the immune-related costs of social status in a wide range of species.

One conceptual framework that may prove useful in future analyses is the idea that status-related variation in *allostatic load*—defined as the cumulative physiological burdens exerted on the body to meet life-history demands [168–170]—may predict status-related differences in immune defence. In 2004, Goymann & Wingfield [166] proposed that differences in allostatic load between high- and low-status individuals should predict status-related differences in glucocorticoid hormone levels. In support, this study found a significant relationship between the relative allostatic load of high- versus low-ranking animals and their relative glucocorticoid levels. This allostatic load framework may be useful to clarify the costs experienced by high and low social status males. Indeed, Goymann & Wingfield's [166] framework incorporates some ideas from the trade-offs model (i.e. the cost of rank acquisition and maintenance), some of the ideas of the stress–response model (i.e. degree of threat from dominants, outlets to avoid conflict), as well as environmental effects (i.e. resource control and availability). To date, Goyman's and Wingfield's approach has not been applied to immune responses or other measures of health, but we think this may be a fruitful approach to develop future predictive models of variation in male health as a function of social status.

### (b) Immune responses versus patterns of parasitism

While we did not observe consistent links between dominance status and immune responses, we did observe a striking correlation between male dominance status and patterns of parasitism. Specifically, dominant males almost always experienced higher parasite prevalence, intensity of infection or parasite species richness than subordinate males. These results are puzzling: why would dominant males experience higher disease risk than subordinates, but demonstrate so few differences in immune responses as compared to subordinates? These findings may be explained by both biases in study setting as well as real biological phenomena.

As discussed previously, most tests of immune response occurred in captive settings, while most measures of parasitism were obtained in semi-natural or wild conditions. Thus, study context may explain the differences in patterns of immune response versus parasitism because captive subjects are often treated for parasites as part of animal care and use policies. Even when treatment is given, captive animals may be exposed to diseases and parasites that are not prevalent in their natural environment [171,172]. Thus, when captive subjects experience parasitism, such patterns may be viewed as animal management problems rather than opportunities to measure differences in parasite infection between individuals.

The observation that dominant males were more parasitized but exhibited few differences in immunity compared with subordinate males may also be caused by real biological phenomena. First, as Lee [31] suggests, differences in social interactions, diet and foraging behaviours may lead to ecological differences between dominant and subordinate males that affect exposure to pathogens [29,30,37]. In support, dominant individuals tend to have priority of access to food (e.g. [111,140,173]) and mates (e.g. [27,174,175]), which may result in differential exposure to parasites. For example, dominant male gazelles, which vigorously defend parasite-rich breeding territories, subsequently exhibit significantly greater gastrointestinal nematode burdens compared with subordinate bachelor males [30]. Furthermore, in feral cats, dominant males have priority of access to mates, larger home ranges and higher rates of feline immunodeficiency virus (FIV) than subordinate males [176].

Second, dominant males may also be more tolerant of parasites than subordinate males [177,178]. Specifically, instead of using immune responses to resist parasite infection, dominant males may bear the burden of parasite infection without experiencing substantial symptoms or compromising their health. The mechanisms that underlie parasite tolerance are not well known, but may include a greater ability to repair tissue damage caused by parasites [178]. In support, in baboons, dominant males appear to heal more quickly from naturally occurring wounds than subordinate males [179]. Future studies that compare status-related differences in MHC variation and its association with tolerance may also shed light on this topic.

Finally, our results suggest that, while dominant and subordinate males largely experience similar patterns of immune response, the costs of high rank may lead dominant males to experience immunosuppression of one aspect of immunity: antibody production in response to antigens. Antibody production plays a key role in parasite resistance [180,181], and suppressed antibody responses may be linked to higher disease risk in dominant males. For instance, Halvorsen [159] found that during the mating season, male reindeer exhibit decreased antibodies to the nematode *Elaphostronylus rangiferi*. Such results may also point to a trade-off between Th-2 and Th-1 mediated defences in dominant males. In particular, antibody production is one aspect of Th-2 mediated defence, and Th-2 defences can downregulate Th-1 defences and vice versa [182]. Interestingly, in the present meta-analytic review, dominant males had significantly greater DTH reactions than subordinates, a measure of Th-1 mediated immunity [79,80]. These results warrant further investigation and provide initial evidence that the higher Th-1 mediated defences found in dominant males may indicate suppression of Th-2-mediated defences, and that the higher Th-2-mediated defences found in subordinate males may indicate suppression of Th-1-mediated defences.

### (c) Methodological issues and future directions

This review illustrates some of the challenges of using meta-analyses to understand status-related differences in health. In particular, 35 papers were excluded simply because authors did not properly report effect sizes and/or sample sizes. This was especially true when studies found non-significant and negative results. In a number of cases, we were able to circumvent this problem by extracting means and estimates of variance from figures. However, we must emphasize that a major shortcoming of many of the published studies we reviewed was the tendency of some authors to overemphasize positive results and to largely ignore non-significant and negative results. This pattern is evident in the trends we observed for publication bias (e.g. electronic supplementary material, figures S7, S8, S15 and S16). Petticrew & Smith [183], in a review of the status-related effects of stress on coronary artery disease, argue that this practice of selectively emphasizing positive results has resulted in inaccurate citations and overstatements of the ‘strength of associations between variables’ [184,185]. Consequently, the sample sizes for our meta-analyses were considerably less than the number of published studies on status-related differences in male immunity and parasitism. Furthermore, our meta-analytic review revealed a lack of studies that measure diverse aspects of immunity in males in wild populations. Only 17% of the studies occurred in wild settings and most of these assessed patterns of parasitism. Importantly, few studies measured both parasitism and immune response in the same system, especially in wild populations. Therefore, we suggest that tests of parasitism are conducted in conjunction with tests of immune response in natural environments (e.g. see [161]). Also, further research on tolerance versus resistance to infection, as well as trade-offs in Th-1 versus Th-2 mediated immunity, may provide new insights. Lastly, we think that a greater focus on the differences in the strength of allostatic load experienced by dominants and subordinates may be a fruitful direction for future research.

## 5. Conclusion

Two paradigms have been used to explain status-related differences in male health, one that predicts immunosuppression in dominant males [23–28], and another that predicts immunosuppression in subordinate males [16,38–40]. The results of the present meta-analyses provided little support for either paradigm. As such, these findings reveal the considerable limitations of current theory and the need for new competing models. Despite glaringly few differences in immunity, dominant males, almost always had higher parasitism than subordinate males including measures of blood parasites, ectoparasites and gastrointestinal helminths. These results indicate the need for further research on the differences in ecological niches between dominant and subordinate males and the interplay between parasite tolerance and susceptibility. Furthermore, one major hurdle to understanding status-related differences in health and immunity is a lack of studies that measure diverse aspects of immunity in males in wild populations. Hence, we support the growing consensus among ecoimmunologists that more immunological studies, addressing a broader range of immune components, need to take place in natural environments in order to better understand adaptive variation in immune function [57,62,186,187].

## Supplementary Material

Supplementary tables and figures

## Data accessibility

The datasets supporting this article have been uploaded as part of the electronic supplementary material. Data used for meta-analyses have been uploaded to the Dryad repository (doi:10.5061/dryad.54s81).

## References

[1] Alexander RD 1974 The evolution of social behaviour. Annu. Rev. Ecol. Syst. 5, 325–383 (10.1146/annurev.es.05.110174.001545)

[2] Cattell RB 1942 The concept of social status. J. Psychosoc. Res. 15, 293–308.

[3] Landtman G 1970 The origin of the inequality of the social classes. London, UK: AMS Press.

[4] Tejerina B Perugorria I Benski T Langman L 2013 From indignation to occupation: a new wave of global mobilization. Curr. Sociol. 61, 377–392. (10.1177/0011392113479738)

[5] Berlet C 2011 Taking tea parties seriously: corporate globalization, populism, and resentment. Perspect. Glob. Dev. Technol. 10, 11–29. (10.1163/156914911X555071)

[6] Antonovsky A 1967 Social class, life expectancy, and overall mortality. Milbank Memorial Fund Q. Health Soc. 45, 31–73. (10.2307/3348839) 6034566

[7] Adler NE Boyce WT Chesney MA Folkman S Syme SL 1993 Socioeconomic inequalities in health—no easy solution. J. Am. Med. Assoc. 269, 3140–3145. (10.1001/jama.1993.03500240084031) 8505817

[8] Syme SL Berkman LF 1976 Social class, susceptibility and sickness. Am. J. Epidemiol. 104, 1–8. 779462 10.1093/oxfordjournals.aje.a112268

[9] Martinez M Calvo-Torrent A Pico-Alfonso MA 1998 Social defeat and subordination as models of social stress in laboratory rodents: a review. Aggress. Behav. 24, 241–256. (10.1002/(SICI)1098-2337(1998)24:4<241::AID-AB1>3.0.CO;2-M)

[10] Sa-Rocha VM Sa-Rocha LC Palermo-Neto J 2006 Variations in behavior, innate immunity and host resistance to B16F10 melanoma growth in mice that present social stable hierarchical ranks. Physiol. Behav. 88, 108–115. (10.1016/j.physbeh.2006.03.015) 16647094

[11] Avitsur R Kinsey SG Bidor K Bailey MT Padgett DA Sheridan JF 2007 Subordinate social status modulates the vulnerability to the immunological effects of social stress. Psychoneuroendocrinology 32, 1097–1105. (10.1016/j.psyneuen.2007.09.005) 17954013 PMC2151960

[12] Gonzalez G Sorci G de Lope F 1999 Seasonal variation in the relationship between cellular immune response and badge size in male house sparrows (*Passer domesticus*). Behav. Ecol. Sociobiol. 46, 117–122. (10.1007/s002650050600)

[13] Casto JM Nolan V Ketterson ED 2001 Steroid hormones and immune function: experimental studies in wild and captive dark-eyed juncos (*Junco hyemalis*). Am. Nat. 157, 408–420. (10.1086/319318) 18707250

[14] Muehlenbein MP 2006 Intestinal parasite infections and fecal steroid levels in wild chimpanzees. Am. J. Phys. Anthropol. 130, 546–550. (10.1002/ajpa.20391) 16444733

[15] Negro SS Caudron AK Dubois M Delahaut P Gemmell NJ 2010 Correlation between male social status, testosterone levels, and parasitism in a dimorphic polygynous mammal. PLoS ONE 5, e12507 (10.1371/journal.pone.0012507) 20856933 PMC2938340

[16] Sapolsky RM 2004 Social status and health in humans and other animals. Annu. Rev. Anthropol. 33, 393–418. (10.1146/annurev.anthro.33.070203.144000)

[17] Marmot M 2004 The status syndrome: how your social standing affects your health and life expectancy. London, UK: Bloomsbury.

[18] Fairbanks B Hawley DM 2011 Interactions between host social behavior, physiology, and disease susceptibility: the role of dominance status and social context. In Ecoimmunology (eds Demas E Nelson R), pp. 440–467. London, UK: Oxford University Press.

[19] Stearns SC 1992 The evolution of life histories. London, UK: Oxford University Press.

[20] Zuk M Johnsen TS 2000 Social environment and immunity in male red jungle fowl. Behav. Ecol. 11, 146–153. (10.1093/beheco/11.2.146)

[21] Rolff J 2002 Bateman's principle and immunity. Proc. R. Soc. Lond. B 269, 867–872. (10.1098/rspb.2002.1959) PMC1690964 11958720

[22] Mills SC Grapputo A Jokinen I Koskela E Mappes T Poikonen T 2010 Fitness trade-offs mediated by immunosuppression costs in a small mammal. Evolution 64, 166–179. (10.1111/j.1558-5646.2009.00820.x) 19686266

[23] Muehlenbein MP Bribiescas RG 2005 Testosterone-mediated immune functions and male life histories. Am. J. Hum. Biol. 17, 527–558. (10.1002/ajhb.20419) 16136532

[24] Folstad I Karter AJ 1992 Parasites, bright males, and the immunocompetence handicap. Am. Nat. 139, 603–622. (10.1086/285346)

[25] Hamilton WD Zuk M 1982 Heritable true fitness and bright birds—a role for parasites. Science 218, 384–387. (10.1126/science.7123238) 7123238

[26] Roberts ML Buchanan KL Hasselquist D Evans MR 2007 Effects of testosterone and corticosterone on immunocompetence in the zebra finch. Horm. Behav. 51, 126–134. (10.1016/j.yhbeh.2006.09.004) 17049519

[27] Mills SC Grapputo A Jokinen I Koskela E Mappes T Oksanen TA Poikonen T 2009 Testosterone-mediated effects on fitness-related phenotypic traits and fitness. Am. Nat. 173, 475–487. (10.1086/597222) 19236274

[28] Muehlenbein MP Watts DP 2010 The costs of dominance: testosterone, cortisol and intestinal parasites in wild male chimpanzees. BioPsychoSoc. Med. 4, 1–12. (10.1186/1751-0759-4-21) 21143892 PMC3004803

[29] Altizer S 2003 Social organization and parasite risk in mammals: integrating theory and empirical studies. Annu. Rev. Ecol. Evol. Syst. 34, 517–547. (10.1146/annurev.ecolsys.34.030102.151725)

[30] Ezenwa VO 2004 Host social behavior and parasitic infection: a multifactorial approach. Behav. Ecol. 15, 446–454. (10.1093/beheco/arh028)

[31] Lee KA 2006 Linking immune defenses and life history at the levels of the individual and the species. Integr. Compar. Biol. 46, 1000–1015. (10.1093/icb/icl049) 21672803

[32] Saino N Moller AP 1996 Sexual ornamentation and immunocompetence in the barn swallow. Behav. Ecol. 7, 227–232. (10.1093/beheco/7.2.227)

[33] Poiani A Goldsmith AR Evans MR 2000 Ectoparasites of house sparrows (*Passer domesticus*): an experimental test of the immunocompetence handicap hypothesis and a new model. Behav. Ecol. Sociobiol. 47, 230–242. (10.1007/s002650050660)

[34] Ressel S Schall JJ 1989 Parasites and showy males—malarial infection and color variation in fence lizards. Oecologia 78, 158–164. (10.1007/BF00377151) 28312354

[35] Muehlenbein MP Alger J Cogswell F James M Krogstad D 2005 The reproductive endocrine response to *Plasmodium vivax* infection in Hondurans. Am. J. Trop. Med. Hyg. 73, 178–187. 16014855

[36] Decristophoris PMA von Hardenberg A McElligott AG 2007 Testosterone is positively related to the output of nematode eggs in male Alpine ibex (*Capra ibex*) faeces. Evol. Ecol. Res. 9, 1277–1292.

[37] Grear DA Perkins SE Hudson PJ 2009 Does elevated testosterone result in increased exposure and transmission of parasites? Ecol. Lett. 12, 528–537. (10.1111/j.1461-0248.2009.01306.x) 19392718

[38] Cohen S Janicki-Deverts D Miller GE 2007 Psychological stress and disease. J. Am. Med. Assoc. 298, 1685–1687. (10.1001/jama.298.14.1685) 17925521

[39] McEwen BS 2008 Central effects of stress hormones in health and disease: understanding the protective and damaging effects of stress and stress mediators. Eur. J. Pharmacol. 583, 174–185. (10.1016/j.ejphar.2007.11.071) 18282566 PMC2474765

[40] Dhabhar FS 2009 A hassle a day may keep the pathogens away: the fight-or-flight stress response and the augmentation of immune function. Integr. Comp. Biol. 49, 215–236. (10.1093/icb/icp045) 21665815

[41] Sapolsky RM 2005 The influence of social hierarchy on primate health. Science 308, 648–652. (10.1126/science.1106477) 15860617

[42] Herbert TB Cohen S 1993 Stress and immunity in humans—a metaanalytic review. Psychosom. Med. 55, 364–379. (10.1097/00006842-199307000-00004) 8416086

[43] Bohus B Koolhaas JM Deruiter AJH Heijnen CJ 1991 Stress and differential alterations in immune system functions—conclusions from social stress studies in animals. Neth. J. Med. 39, 306–315. 1791892

[44] Tamashiro KLK Nguyen MMN Sakai RR 2005 Social stress: from rodents to primates. Front. Neuroendocrinol. 26, 27–40. (10.1016/j.yfrne.2005.03.001) 15862183

[45] Bartolomucci A 2005 Resource loss and stress-related disease: is there a link? Med. Sci. Monitor. 11, RA147–RA154. 15874905

[46] Avitsur R Padgett DA Sheridan JF 2006 Social interactions, stress, and immunity. Neurol. Clin. 24, 483–491. (10.1016/j.ncl.2006.03.005) 16877119

[47] Bartolomucci A 2007 Social stress., immune functions and disease in rodents. Front. Neuroendocrinol. 28, 28–49. (10.1016/j.yfrne.2007.02.001) 17379284

[48] Cavigelli SA Chaudhry HS 2012 Social status, glucocorticoids, immune function, and health: can animal studies help us understand human socioeconomic-status-related health disparities? Horm. Behav. 62, 295–313. (10.1016/j.yhbeh.2012.07.006) 22841799

[49] Smith A Vollmer-Conna U Bennett B Wakefield D Hickie I Lloyd A 2004 The relationship between distress and the development of a primary immune response to a novel antigen. Brain Behav. Immunol. 18, 65–75. (10.1016/S0889-1591(03)00107-7) 14651948

[50] Marshall GD Agarwal SK 2000 Stress, immune regulation, and immunity: applications for asthma. Allergy Asthma Proc. 21, 241–246. (10.2500/108854100778248917) 10951892

[51] Mosmann TR Sad S 1996 The expanding universe of T-cell subsets: Th1, Th2 and more. Immunol. Today 17, 138–146. (10.1016/0167-5699(96)80606-2) 8820272

[52] Zhou DH Kusnecov AW Shurin MR Depaoli M Rabin BS 1993 Exposure to physical and psychological stressors elevates plasma interleukin-6—relationship to the activation of the hypothalamic–pituitary–adrenal axis. Endocrinology 133, 2523–2530. 8243274 10.1210/endo.133.6.8243274

[53] Glaser R Kiecolt-Glaser JK 2005 Science and society—stress-induced immune dysfunction: implications for health. Nat. Rev. Immunol. 5, 243–251. (10.1038/nri1571) 15738954

[54] Braude S Tang-Martinez Z Taylor GT 1999 Stress, testosterone, and the immunoredistribution hypothesis. Behav. Ecol. 10, 345–350. (10.1093/beheco/10.3.345)

[55] Roberts ML Buchanan KL Evans MR 2004 Testing the immunocompetence handicap hypothesis: a review of the evidence. Anim. Behav. 68, 227–239. (10.1016/j.anbehav.2004.05.001)

[56] Adamo SA 2004 How should behavioural ecologists interpret measurements of immunity? Anim. Behav. 68, 1443–1449. (10.1016/j.anbehav.2004.05.005)

[57] Martin LB Weil ZM Nelson RJ 2006 Refining approaches and diversifying directions in ecoimmunology. Integr. Comp. Biol. 46, 1030–1039. (10.1093/icb/icl039) 21672805

[58] Schmid-Hempel P Ebert D 2003 On the evolutionary ecology of specific immune defence. Trends Ecol. Evol. 18, 27–32. (10.1016/S0169-5347(02)00013-7)

[59] Sheldon BC Verhulst S 1996 Ecological immunology: costly parasite defences and trade-offs in evolutionary ecology. Trends Ecol. Evol. 11, 317–321. (10.1016/0169-5347(96)10039-2) 21237861

[60] Matthews KA Gallo LC 2011 Psychological perspectives on pathways linking socioeconomic status and physical health. Annu. Rev. Psychol. 62, 501–530. (10.1146/annurev.psych.031809.130711) 20636127 PMC3121154

[61] Watts DP 2010 Dominance, power, and politics in nonhuman and human primates. In Mind the gap: tracing the origins of human universals (eds Kappeler P Silk JB), pp. 109–138. New York, NY: Springer.

[62] Nunn C Altizer S 2006 Infectious diseases in primates: behavior, ecology, and evolution. London, UK: Oxford University Press.

[63] Sompayrac L 2012 How the immune system works. Oxford, UK: John Wiley & Sons.

[64] Kindt TJ Goldsby RA Osborne BA Kuby J 2007 Kuby immunology. New York, NY: Macmillan.

[65] Tieleman BI Williams JB Ricklefs RE Klasing KC 2005 Constitutive innate immunity is a component of the pace-of-life syndrome in tropical birds. Proc. R. Soc. B. 272, 1715–1720. (10.1098/rspb.2005.3155) PMC1559858 16087427

[66] Millet S Bennett J Lee KA Hau M Klasing KC 2007 Quantifying and comparing constitutive immunity across avian species. Dev. Comp. Immunol. 31, 188–201. (10.1016/j.dci.2006.05.013) 16870251

[67] Powrie F Coffman RL 1993 Cytokine regulation of T-cell function—potential for therapeutic intervention. Immunol. Today 14, 270–274. (10.1016/0167-5699(93)90044-L) 8104408

[68] Elenkov IJ Chrousos GP 1999 Stress hormones, Th1/Th2 patterns, pro/anti-inflammatory cytokines and susceptibility to disease. Trends Endocrinol. Metab. 10, 359–368. (10.1016/S1043-2760(99)00188-5) 10511695

[69] Kidd P 2003 Th1/Th2 balance: the hypothesis, its limitations, and implications for health and disease. Altern. Med. Rev. 8, 223–246. 12946237

[70] Elenkov IJ 2004 Glucocorticoids and the Th1/Th2 balance. Glucocorticoid action: basic and clinical implications. Ann. NY Acad. Sci. 1024, 138–146. (10.1196/annals.1321.010) 15265778

[71] Sapolsky RM 1995 Social subordinance as a marker of hypercortisolism—some unexpected subtleties. Ann. NY Acad. Sci. 771, 626–639. (10.1111/j.1749-6632.1995.tb44715.x) 8597436

[72] Glaser R MacCallum RC Laskowski BF Malarkey WB Sheridan JF Kiecolt-Glaser JK 2001 Evidence for a shift in the Th-1 to Th-2 cytokine response associated with chronic stress and aging. J. Gerontol. 56, M477–M482. (10.1093/gerona/56.8.M477) 11487599

[73] Rohatgi A 2014 WebPlotDigitizer 3.3. See http://arohatgi.info/WebPlotDigitizer

[74] Lipsey MW Wilson DB 2002 Effect size calculator and SPSS macros. See http://mason.gmu.edu/~dwilsonb/ma.html

[75] Del Re AC 2013 compute.es: compute effect sizes. R Package See http://cran.r-project.org/web/packages/compute.es

[76] R Development Core Team. 2012 R: a language and environment for statistical computing. Vienna, Austria: R Foundation for Statistical Computing.

[77] Cahan S Gamliel E 2011 First among others? Cohen's d vs. alternative standardized mean group difference measures. Pract. Assess. Res. Eval. 16, 1–10.

[78] Viechtbauer W 2010 Conducting meta-analyses in R with the metafor Package. J. Stat. Softw. 36, 1–48.

[79] Boughton RK Joop G Armitage SAO 2011 Outdoor immunology: methodological considerations for ecologists. Funct. Ecol. 25, 81–100. (10.1111/j.1365-2435.2010.01817.x)

[80] Demas GE Zysling DA Beechler BR Muehlenbein MP French SS 2011 Beyond phytohaemagglutinin: assessing vertebrate immune function across ecological contexts. J. Anim. Ecol. 80, 710–730. (10.1111/j.1365-2656.2011.01813.x) 21401591

[81] Egger M Smith GD Schneider M Minder C 1997 Bias in meta-analysis detected by a simple, graphical test. Br. Med. J. 315, 629–634. (10.1136/bmj.315.7109.629) 9310563 PMC2127453

[82] Smith FV Barnard CJ Behnke JM 1996 Social odours, hormone modulation and resistance to disease in male laboratory mice, *Mus musculus*. Anim. Behav. 52, 141–153. (10.1006/anbe.1996.0160)

[83] Barnard CJ Behnke JM Sewell J 1993 Social behavior, stress and susceptibility to infection in house mice (*Mus musculus*)—effects of duration of grouping and aggressive behavior prior ro infection on susceptibility to *Babesia microti*. Parasitology 107, 183–192. (10.1017/S0031182000067299) 8414673

[84] Barnard CJ Behnke JM Sewell J 1994 Social behavior and susceptibility to infection in house mice (*Mus musculus*)—effects of group size, aggressive behavior and status-related hormonal responses prior to infection on resistance to *Babesia microti*. Parasitology 108, 487–496. (10.1017/S0031182000077349) 8052503

[85] Barnard CJ Behnke JM Sewell J 1996 Social status and resistance to disease in house mice (*Mus musculus*): status-related modulation of hormonal responses in relation to immunity costs in different social and physical environments. Ethology 102, 63–84. (10.1111/j.1439-0310.1996.tb01104.x)

[86] Barnard CJ Behnke JM Gage AR Brown H Smithurst PR 1997 Modulation of behaviour and testosterone concentration in immunodepressed male laboratory mice (*Mus musculus*). Physiol. Behav. 61, 907–917. (10.1016/S0031-9384(97)00011-5) 9177566

[87] Cunnick JE Cohen S Rabin BS Carpenter AB Manuck SB Kaplan JR 1991 Alterations in specific antibody production due to rank and social instability. Brain Behav. Immunol. 5, 357–369. (10.1016/0889-1591(91)90031-5) 1777730

[88] Dijkstra PD Wiegertjes GF Forlenza M van der Sluijs I Hofmann HA Metcalfe NB Groothhuis GG 2011 The role of physiology in the divergence of two incipient cichlid species. J. Evol. Biol. 24, 2639–2652. (10.1111/j.1420-9101.2011.02389.x) 21955260

[89] Fauman MA 1987 The relation of dominant and submissive behavior to the humoral immune response in BALB/C mice. Biol. Psychiatr. 22, 776–779. (10.1016/0006-3223(87)90211-3) 3593816

[90] Hinson ER Hannah MF Norris DE Glass GE Klein SL 2006 Social status does not predict responses to Seoul virus infection or reproductive success among male Norway rats. Brain Behav. Immunol. 20, 182–190. (10.1016/j.bbi.2005.06.003) PMC4128169 16040226

[91] Iglauer F Deutsch W Gartner K Schwarz GO 1992 The influence of genotypes and social ranks in the clinical course of an experimental infection with *Mycoplasma pulmonis* (MRM) in inbred rats. J. Vet. Med. B 39, 672–682. (10.1111/j.1439-0450.1992.tb01221.x) 1492524

[92] Lindstrom KM Hasselquist D Wikelski M 2005 House sparrows (*Passer domesticus*) adjust their social status position to their physiological costs. Horm. Behav. 48, 311–320. (10.1016/j.yhbeh.2005.04.002) 15896793

[93] Saks L Ots I Horak P 2003 Carotenoid-based plumage coloration of male greenfinches reflects health and immunocompetence. Oecologia 134, 301–307. (10.1007/s00442-002-1125-z) 12647136

[94] Vekovishcheva OI Sukhotina IA Zvartau EE 1998 Co-housing in the group with stable hierarchy is not aversive for dominant and subordinate animals. Neurosci. Behav. Physiol. 30, 195–200. (10.1007/BF02463158) 10872730

[95] Bartolomucci A Palanza P Gaspani L Limiroli E Panerai AE Ceresini G Poli MD Parmigiani S 2001 Social status in mice: behavioral, endocrine and immune changes are context dependent. Physiol. Behav. 73, 401–410. (10.1016/S0031-9384(01)00453-X) 11438368

[96] Bartolomucci A Palanza P Sacerdote P Ceresini G Chirieleison A Panerai AE Parmigiani S 2003 Individual housing induces altered immunoendocrine responses to psychological stress in male mice. Psychoneuroendocrinology 28, 540–558. (10.1016/S0306-4530(02)00039-2) 12689611

[97] Bohus B Koolhaas JM Heijnen CJ Deboer O 1993 Immunological responses to social stress—dependence on social environment and coping abilities. Neuropsychobiology 28, 95–99. (10.1159/000119008) 8255418

[98] Cacho R Fano E Areso P Garmendia L Vegas O Brain PF Azpiroz A 2003 Endocrine and lymphoproliferative response changes produced by social stress in mice. Physiol. Behav. 78, 505–512. (10.1016/S0031-9384(03)00018-0) 12676288

[99] Devoino L Alperina E Kudryavtseva N Popova N 1993 Immune responses in male mice with aggressive and submissive behavior patterns—strain differences. Brain Behav. Immunol. 7, 91–96. (10.1006/brbi.1993.1009) 8471801

[100] Hardy CA Quay J Livnat S Ader R 1990 Altered lymphocyte T-response following aggressive encounters in mice. Physiol. Behav. 47, 1245–1251. (10.1016/0031-9384(90)90378-H) 2395930

[101] Kaplan JR Heise ER Manuck SB Shively CA Cohen S Rabin BS Kasprowicz AL 1991 The relationship of agonistic and affiliative behavior patterns to cellular immune function among cynomolgus monkeys (*Macaca fascicularis*) living in unstable social groups. Am. J. Primatol. 25, 157–173. (10.1002/ajp.1350250303) 31948179

[102] Raab A Dantzer R Michaud B Mormede P Taghzouti K Simon H Le Moal M 1986 Behavioral, physiological, and immunological consequences of social status and aggression in chronically coexisting resident-intruder dyads of male rats. Physiol. Behav. 36, 223–228. (10.1016/0031-9384(86)90007-7) 3960994

[103] Stefanski V 1998 Social stress in loser rats: opposite immunological effects in submissive and subdominant males. Physiol. Behav. 63, 605–613. (10.1016/S0031-9384(97)00492-7) 9523905

[104] Stefanski V 2001 Social stress in laboratory rats—behavior, immune function, and tumor metastasis. Physiol. Behav. 73, 385–391. (10.1016/S0031-9384(01)00495-4) 11438366

[105] Stefanski V Engler H 1999 Social stress, dominance and blood cellular immunity. J. Neuroimmunol. 94, 144–152. (10.1016/S0165-5728(98)00242-2) 10376947

[106] Zuk M Johnsen TS 1998 Seasonal changes in the relationship between ornamentation and immune response in red jungle fowl. Proc. R. Soc. Lond. B 265, 1631–1635. (10.1098/rspb.1998.0481)

[107] Pryke SR Astheimer LB Buttemer WA Griffith SC 2007 Frequency-dependent physiological trade-offs between competing colour morphs. Biol. Lett. 3, 494–497. (10.1098/rsbl.2007.0213) 17609174 PMC2391178

[108] Beden SN Brain PF 1985 The primary immune responses to sheep red blood cells in mice of differing social rank or from individual housing. IRCS Med. Sci. 13, 364–365.

[109] Barnard CJ Behnke JM Gage AR Brown H Smithurst PR 1998 The role of parasite-induced immunodepression, rank and social environment in the modulation of behaviour and hormone concentration in male laboratory mice (*Mus musculus*). Proc. R. Soc. Lond. B 265, 693–701. (10.1098/rspb.1998.0349) PMC1689026 9608729

[110] Stefanski V Hendrichs H 1996 Social confrontation in male guinea pigs: behavior, experience, and complement activity. Physiol. Behav. 60, 235–241. (10.1016/0031-9384(95)02269-4) 8804669

[111] Cammarata M Vazzana M Accardi D Parrinello N 2012 Seabream (*Sparus aurata*) long-term dominant–subordinate interplay affects phagocytosis by peritoneal cavity cells. Brain Behav. Immunol. 26, 580–587. (10.1016/j.bbi.2012.01.008) 22289430

[112] Azpiroz A Arregui A Fano E Garmendia L Sanchezmartin JR 1994 Fighting experiences and natural killer activity in male laboratory mice. Aggress. Behav. 20, 67–72. (10.1002/1098-2337(1994)20:1<67::AID-AB2480200108>3.0.CO;2-D)

[113] Wrona D Sukiennik L Jurkowski MK Jurkowlaniec E Glac W Tokarski J 2005 Effects of amphetamine on NK-related cytotoxicity in rats differing in locomotor reactivity and social position. Brain Behav. Immunol. 19, 69–77. (10.1016/j.bbi.2004.04.002) 15581740

[114] Filby AL Paull GC Bartlett EJ Van Look KJW Tyler CR 2010 Physiological and health consequences of social status in zebrafish (*Danio rerio*). Physiol. Behav. 101, 576–587. (10.1016/j.physbeh.2010.09.004) 20851709

[115] Steptoe A Owen N Kunz-Ebrecht S Mohamed-Ali V 2002 Inflammatory cytokines, socioeconomic status, and acute stress responsivity. Brain Behav. Immunol. 16, 774–784. (10.1016/S0889-1591(02)00030-2) 12480506

[116] Merlot E Moze E Bartolomucci A Dantzer R Neveu PJ 2004 The rank assessed in a food competition test influences subsequent reactivity to immune and social challenges in mice. Brain Behav. Immunol. 18, 468–475. (10.1016/j.bbi.2003.11.007) 15265540

[117] Savignac HM Hyland NP Dinan TG Cryan JF 2011 The effects of repeated social interaction stress on behavioural and physiological parameters in a stress-sensitive mouse strain. Behav. Brain Res. 216, 576–584. (10.1016/j.bbr.2010.08.049) 20826188

[118] Audet MC Mangano EN Anisman H 2010 Behavior and pro-inflammatory cytokine variations among submissive and dominant mice engaged in aggressive encounters: moderation by corticosterone reactivity. Front. Behav. Neurosci. 4, 156 (10.3389/fnbeh.2010.00156) 20838478 PMC2936936

[119] Fano E Sanchez-Martin JR Arregi A Castro B Alonso A Brain P Azpiroz A 2001 Social stress paradigms in male mice: variations in behavior, stress and immunology. Physiol. Behav. 73, 165–173. (10.1016/S0031-9384(01)00445-0) 11399308

[120] Bonamin LV Malucelli BE 1995 The influence of social isolation and peripheral innervation on Ehrlich tumor growth in mice. Braz. J. Med. Biol. Res. 28, 557–562. 8555976

[121] Cohen S Line S Manuck SB Rabin BS Heise ER Kaplan JR 1997 Chronic social stress, social status, and susceptibility to upper respiratory infections in nonhuman primates. Psychosom. Med. 59, 213–221. (10.1097/00006842-199705000-00001) 9254393

[122] Grimm MS Emerman JT Weinberg J 1996 Effects of social housing condition and behavior on growth of the Shionogi mouse mammary carcinoma. Physiol. Behav. 59, 633–642. (10.1016/0031-9384(95)02126-4) 8778846

[123] Kavelaars A Heijnen CJ Tennekes R Bruggink JE Koolhaas JM 1999 Individual behavioral characteristics of wild-type rats predict susceptibility to experimental autoimmune encephalomyelitis. Brain Behav. Immunol. 13, 279–286. (10.1006/brbi.1998.0534) 10600216

[124] Lindstrom KM 2004 Social status in relation to Sindbis virus infection clearance in greenfinches. Behav. Ecol. Sociobiol. 55, 236–241. (10.1007/s00265-003-0703-3)

[125] Lindstrom K Lundstrom J 2000 Male greenfinches (*Carduelis chloris*) with brighter ornaments have higher virus infection clearance rate. Behav. Ecol. Sociobiol. 48, 44–51. (10.1007/s002650000217)

[126] Padgett DA Sheridan JF Dorne J Berntson GG Candelora J Glaser R 1998 Social stress and the reactivation of latent herpes simplex virus type 1. Proc. Natl Acad. Sci USA 95, 7231–7235. (10.1073/pnas.95.12.7231) 9618568 PMC22787

[127] Sheridan JF Stark JL Avitsur R Padgett DA 2000 Social disruption, immunity, and susceptibility to viral infection—role of glucocorticoid insensitivity and NGF. Ann. NY Acad. Sci. 917, 894–905. (10.1111/j.1749-6632.2000.tb05455.x) 11270350

[128] Barnum CJ Blandino P Jr Deak T 2008 Social status modulates basal IL-1 concentrations in the hypothalamus of pair-housed rats and influences certain features of stress reactivity. Brain Behav. Immunol. 22, 517–527. (10.1016/j.bbi.2007.10.004) 18037266

[129] Gartner K Kirchhoff H Mensing K Velleuer R 1989 The influence of social rank on the susceptibility of rats to *Mycoplasma arthritidis*. J. Behav. Med. 12, 487–502. (10.1007/BF00844880) 2614823

[130] Stefanski V 2000 Social stress in laboratory rats: hormonal responses and immune cell distribution. Psychoneuroendocrinology 25, 389–406. (10.1016/S0306-4530(99)00066-9) 10725615

[131] Liljedal S Folstad I 2003 Milt quality, parasites, and immune function in dominant and subordinate Arctic charr. Can. J. Zool. 81, 221–227. (10.1139/z02-244)

[132] Sapolsky RM 1993 Endocrinology alfresco—psychoendocrine studies of wild baboons. Recent Prog. Horm. Res. 48, 437–468. (10.1016/B978-0-12-571148-7.50020-8) 8441854

[133] Alberts SC Sapolsky RM Altmann J 1992 Behavioral, endocrine, and immunological correlates of immigration by an aggressive male into a natural primate group. Horm. Behav. 26, 167–178. (10.1016/0018-506X(92)90040-3) 1612563

[134] Dufva R Allander K 1995 Intraspecific variation in plumage coloration reflects immune response in Great Tit (*Parsus major*). Funct. Ecol. 9, 785–789. (10.2307/2390253)

[135] Zuk M Johnsen TS Maclarty T 1995 Endocrine–immune interactions, ornaments and mate choice in red jungle fowl. Proc. R. Soc. Lond. B 260, 205–210. (10.1098/rspb.1995.0081)

[136] Lopuch S Matula B 2008 Is there a relationship between dominance rank and condition in captive male bank voles, *Clethrionomys glareolus*? Acta Ethol. 11, 1–5. (10.1007/s10211-007-0035-9)

[137] Blanchard DC Spencer RL Weiss SM Blanchard RJ McEwen B Sakai RR 1995 Visible burrow system as a model of chronic social stress—behavioral and neuroendocrine correlates. Psychoneuroendocrinology 20, 117–134. (10.1016/0306-4530(94)E0045-B) 7899533

[138] Brain PF 1972 Endocrine and behavioral differences between dominant and subordinate male house mice housed in pairs. Psychon. Sci. 28, 260–262. (10.3758/BF03328732)

[139] Spencer RL Miller AH Moday H McEwen BS Blanchard RJ Blanchard DC Sakai RR 1996 Chronic social stress produces reductions in available splenic type II corticosteroid receptor binding and plasma corticosteroid binding globulin levels. Psychoneuroendocrinology 21, 95–109. (10.1016/0306-4530(95)00020-8) 8778907

[140] Li F-H Zhong W-Q Wang Z Wang D-H 2007 Rank in a food competition test and humoral immune functions in male Brandt's voles (*Lasiopodomys brandtii*). Physiol. Behav. 90, 490–495. (10.1016/j.physbeh.2006.10.009) 17141282

[141] Turney TH Harmsen AG 1984 Splenomegaly and other hematological parameters in the socially dominant mouse. Physiol. Behav. 33, 559–562. (10.1016/0031-9384(84)90371-8) 6543009

[142] Huyghe K Husak JF Herrel A Tadic Z Moore IT Van Damme R Vanhooydonck B 2009 Relationships between hormones, physiological performance and immunocompetence in a color-polymorphic lizard species, *Podarcis melisellensis*. Horm. Behav. 55, 488–494. (10.1016/j.yhbeh.2009.02.005) 19265697

[143] Mooring MS McKenzie AA Hart BL 1996 Role of sex and breeding status in grooming and total tick load of impala. Behav. Ecol. Sociobiol. 39, 259–266. (10.1007/s002650050288)

[144] Caine J Melfi V 2006 A longitudinal study into the factors affecting the prevalence and intensity of *Trichuris trichiura* in captive *Colobus guereza*. Ratel 33, 5–10.

[145] Halvorsen O 1986 On the relationship between social status of host and risk of parasitic infection. Oikos 47, 71–74. (10.2307/3565921)

[146] Hausfater G Watson DF 1976 Social and reproductive correlates of parasite ova emissions by baboons. Nature 262, 688–689. (10.1038/262688a0) 822345

[147] Kessler MJ Yarbrough B Rawlins RG Berard J 1984 Intestinal parasites of the free-ranging Cayo Santiago rhesus monkeys (*Macaca mulatta*). J. Med. Primatol. 13, 57–66. 6502683

[148] Meade BJ 1983 Host–parasite dynamics among Amboseli baboons (*Papio cynocephalus*). PhD thesis. Virginia Polytechnic Institute and State University, Virginia, USA.

[149] Melfi V Poyser F 2007 *Trichuris* burdens in zoo-housed *Colobus guereza*. Int. J. Primatol. 28, 1449–1456. (10.1007/s10764-007-9206-9)

[150] Poyser F 2003 Social effects on the *Trichuris* burdens of Abyssinian colobus (*Colobus guereza*) housed at Paignton Zoo. In Proc. Fifth Annual Symp. on Zoo Research, Marwell Zoological Park, 7th and 8th July 2003, vol. 5, pp. 126–132.

[151] Teichroeb JA Kutz SJ Parkar U Thompson RCA Sicotte P 2009 Ecology of the gastrointestinal parasites of *Colobus vellerosus* at Boabeng-Fiema, Ghana: Possible anthropozoonotic transmission. Am. J. Phys. Anthropol. 140, 498–507. (10.1002/ajpa.21098) 19434756

[152] Wanless S Barton TR Harris MP 1997 Blood hematocrit measurements of 4 species of North Atlantic seabirds in relation to levels of infestation by the tick *Ixodes uriae*. Colon. Waterbirds 20, 540–544. (10.2307/1521606)

[153] Elias D Wolff K Klassen P Bulux J Solomons NW 1997 Intestinal helminths and their influence on the indicators of iron status in the elderly. J. Nutr. Health Aging 1, 167–173. 10995086

[154] Jones TR 2002 Anemia in parasite- and recombinant protein-immunized *Aotus* monkeys infected with *Plasmodium falciparum*. Am. J. Trop. Med. Hyg. 66, 672–679. 12224573 10.4269/ajtmh.2002.66.672

[155] Kaplan JR Manuck SB Clarkson TB Lusso FM Taub DM 1982 Social status, environment, and atherosclerosis in cynomolgus monkeys. Arteriosclerosis 2, 359–368. (10.1161/01.ATV.2.5.359) 6889852

[156] Mitani JC Watts DP Muller MN 2002 Recent developments in the study of wild chimpanzee behavior. Evol. Anthropol. 11, 9–25. (10.1002/evan.10008)

[157] Richner H Christe P Oppliger A 1995 Paternal investment affects prevalence of malaria. Proc. Natl Acad. Sci. USA 92, 1192–1194. (10.1073/pnas.92.4.1192) 7862659 PMC42664

[158] Buchanan KL Evans MR Goldsmith AR 2003 Testosterone, dominance signalling and immunosuppression in the house sparrow, *Passer domesticus*. Behav. Ecol. Sociobiol. 55, 50–99. (10.1007/s00265-003-0682-4)

[159] Halvorsen O Skorping A Hansen K 1985 Seasonal cycles in the output of 1st-stage larva of the nematode *Elaphostrongylus rangiferi* from reindeer, *Rangifer tarandus tarandus*. Polar Biol. 5, 49–54. (10.1007/BF00446045)

[160] Mysterud A Langvatn R Stenseth NC 2004 Patterns of reproductive effort in male ungulates. J. Zool. 264, 209–215. (10.1017/S0952836904005618)

[161] Ezenwa VO Ekernas LS Creel S 2012 Unravelling complex associations between testosterone and parasite infection in the wild. Funct. Ecol. 26, 123–133. (10.1111/j.1365-2435.2011.01919.x)

[162] Sapolsky RM Spencer EM 1997 Insulin-like growth factor I is suppressed in socially subordinate male baboons. Am. J. Physiol. Reg. I. 273, R1346–R1351. 10.1152/ajpregu.1997.273.4.R1346 9362298

[163] Creel S 2001 Social dominance and stress hormones. Trends Ecol. Evol. 16, 491–497. (10.1016/S0169-5347(01)02227-3)

[164] Buehler DM Piersma T Tieleman BI 2008 Captive and free-living red knots *Calidris canutus* exhibit differences in non-induced immunity that suggest different immune strategies in different environments. J. Avian Biol. 39, 560–566. (10.1111/j.0908-8857.2008.04408.x)

[165] Martin LB Kidd L Liebl AL Coon CAC 2011 Captivity induces hyper-inflammation in the house sparrow (*Passer domesticus*). J. Exp. Biol. 214, 2579–2585. (10.1242/jeb.057216) 21753052

[166] Goymann W Wingfield JC 2004 Allostatic load, social status and stress hormones: the costs of social status matter. Anim. Behav. 67, 591–602. (10.1016/j.anbehav.2003.08.007)

[167] Klein SL 2000 Hormones and mating system affect sex and species differences in immune function among vertebrates. Behav. Process. 51, 149–166. (10.1016/S0376-6357(00)00125-X) 11074318

[168] McEwen BS Stellar E 1993 Stress and the individual—mechanisms leading to disease. Arch. Intern. Med. 153, 2093–2101. (10.1001/archinte.1993.00410180039004) 8379800

[169] Seeman TE McEwen BS Rowe JW Singer BH 2001 Allostatic load as a marker of cumulative biological risk: MacArthur studies of successful aging. Proc. Natl Acad. Sci. USA 98, 4770–4775. (10.1073/pnas.081072698) 11287659 PMC31909

[170] Seeman T Epel E Gruenewald T Karlamangla A McEwen BS 2010 Socioeconomic differentials in peripheral biology: cumulative allostatic load. Ann. NY Acad. Sci. 1186, 223–239. (10.1111/j.1749-6632.2009.05341.x) 20201875

[171] Lim YAL Ngui R Shukri J Rohela M Naim HRM 2008 Intestinal parasites in various animals at a zoo in Malaysia. Vet. Parasitol. 157, 154–159. (10.1016/j.vetpar.2008.07.015) 18723289

[172] Cunningham AA 1996 Disease risks of wildlife translocations. Conserv. Biol. 10, 349–353. (10.1046/j.1523-1739.1996.10020349.x)

[173] Janson C 1985 Aggressive competition and individual food consumption in wild capuchin monkeys (*Cebus apella*). Behav. Ecol. Sociobiol. 18, 125–138. (10.1007/BF00299041)

[174] Muller MN Mitani JC 2005 Conflict and cooperation in wild chimpanzees. Adv. Stud. Behav. 35, 275–331. (10.1016/S0065-3454(05)35007-8)

[175] Natoli E Schmid M Say L Pontier D 2007 Male reproductive success in a social group of urban feral cats (*Felis catus L*.). Ethology 113, 283–289. (10.1111/j.1439-0310.2006.01320.x)

[176] Natoli E Say L Cafazzo S Bonanni R Schmid M Pontier D 2005 Bold attitude makes male urban feral domestic cats more vulnerable to Feline Immunodeficiency Virus. Neurosci. Biobehav. Rev. 29, 151–157. (10.1016/j.neubiorev.2004.06.011) 15652262

[177] Medzhitov R Schneider DS Soares MP 2012 Disease tolerance as a defense strategy. Science 335, 936–941. (10.1126/science.1214935) 22363001 PMC3564547

[178] Hayward AD Nussey DH Wilson AJ Berenos C Pilkington JG Watt KA Pemberton JM Graham AL 2014 Natural selection on individual variation in tolerance of gastrointestinal nematode infection. PLoS Biol. 12, 1–13. (10.1371/journal.pbio.1001917) PMC4114752 25072883

[179] Archie EA Altmann J Alberts SC 2012 Social status predicts wound healing in wild baboons. Proc. Natl Acad. Sci. USA 109, 9017–9022. (10.1073/pnas.1206391109) 22615389 PMC3384186

[180] Moreau E Chauvin A 2010 Immunity against helminths: interactions with the host and the intercurrent infections. J. Biomed. Biotechnol. 2010, 428593 (10.1155/2010/428593) 20150967 PMC2817558

[181] Allen JE Maizels RM 2011 Diversity and dialogue in immunity to helminths. Nat. Rev. Immunol. 11, 375–388. (10.1038/nri2992) 21610741

[182] Neurath MF Finotto S Glimcher LH 2002 The role of Th1/Th2 polarization in mucosal immunity. Nat. Med. 8, 567–573. (10.1038/nm0602-567) 12042806

[183] Petticrew M Smith GD 2012 The monkey puzzle: a systematic review of studies of stress, social hierarchies, and heart disease in monkeys. PLoS ONE 7, e27939 (10.1371/journal.pone.0027939) 22470414 PMC3309950

[184] Sterne JAC Egger M Smith GD 2001 Systematic reviews in health care—investigating and dealing with publication and other biases in meta-analysis. Br. Med. J. 323, 101–105. (10.1136/bmj.323.7304.101) 11451790 PMC1120714

[185] Cummins S Macintyre S 2002 ‘Food deserts’—evidence and assumption in health policy making. Br. Med. J. 325, 436–438. (10.1136/bmj.325.7361.436) 12193363 PMC1123946

[186] Hawley DM Altizer SM 2011 Disease ecology meets ecological immunology: understanding the links between organismal immunity and infection dynamics in natural populations. Funct. Ecol. 25, 48–60. (10.1111/j.1365-2435.2010.01753.x)

[187] Pedersen AB Babayan SA 2011 Wild immunology. Mol. Ecol. 20, 872–880. (10.1111/j.1365-294X.2010.04938.x) 21324009

