# Supplementary material for: Social status, immune response and parasitism in males: a meta-analysis
Source: Philos Trans R Soc Lond B Biol Sci. 2015 May 26;370(1669):20140109. doi: 10.1098/rstb.2014.0109 (PMC4410375; doi:10.1098/rstb.2014.0109)
Supplement: Supplementary tables and figures [file rstb20140109supp1.pdf]

Habig, B and Archie, EA. 2015 Social status, immune response, and parasitism in males: a meta-analysis. *Phil. Trans. R. Soc. B.* **370** doi: 10.1098/rstb.2014.0109

Supplementary materials

Table S1. Methods used to measure male dominance in the 77 studies included in our meta-analyses; note that some studies used multiple methods to assess dominance.

tests of dominance	
behavioral tests	75
food competition	6
outcomes of agonistic interactions	56
territoriality	3
mating behavior	5
investigatory behavior	3
vocalizations	1
sensory contact	1
morphological tests	10
body mass	1
coloration	7
comb size	2
physiological tests	2
testosterone levels	1
weight loss	1
tests of condition	5
coat condition	1
scars/marks	3
wounding index	1
resources as a proxy for dominance	1
employment grade	1

Table S2. Immune assays used to assess the effects of social status on immune function (page 1 of 8)

test of immune function	description of test	what the test measures	immune system components (see Table 1)	predicted direction (tradeoffs model)	predicted direction (stress-response model)	references
baseline immunoglobulins (IgA, IgG, IgE)[A].	use ELISA to measure the concentration of immunoglobulins in plasma in healthy animals.	measures the immune system's state of readiness to deploy antibody-mediated defenses	adaptive constitutive Th-2	conflicting predictions	conflicting predictions	(1, 2)
broad (e.g., total IgG or IgA) or specific antibody response to antigen challenge (e.g. sheep red blood cells, tetanus)	use ELISA to measure the concentration of immunoglobulins in plasma after the animal has been exposed to an antigen	measures adaptive humoral response from B-cells and Th2 cells	adaptive inducible Th-2	D>S	D>S	(1, 2)
in vitro lymphocyte proliferation to a mitogen<br><br>in vitro lymphocyte proliferation to concanavalin A (Con A)<br><br>in vitro lymphocyte proliferation to phytohemagglutinin (PHA)	collect whole blood or remove lymphoid tissue (e.g. spleen) and grind between slides; expose blood/tissue to an antigen and use radioactively labeled thymidine (or other methods) to measure the rate of lymphocyte cell division (i.e. proliferation) in response to the antigen	measures the ability of T and/or B-cells to proliferate in response to an antigen/mitogen; ConA and PHA serve as T-cell mitogens, LPS stimulates B cells predominately, and pokeweed mitogen is a mixed B- and T-cell mitogen	adaptive inducible	D>S	conflicting predictions	(2, 3)

[A] Note, this is relatively non-specific if only broad categories of immunoglobulins (e.g., total IgA or IgG) are measured.

Table S2. Immune assays used to assess the effects of social status on immune function (page 2 of 8)

test of immune function	description of test	what the test measures	immune system components (see Table 1)	predicted direction (tradeoffs model)	predicted direction (stress-response model)	references
skin swelling test after initial exposure. Also called delayed-type hypersensitivity (DTH). Measured in response to antigens/mitogens like PHA, ConA, KLH and DNFB.	inject skin with an antigen to which the animal has previously been exposed. Measure the size of the resulting skin swelling.	predominantly T-cell-mediated responses resulting in the recruitment/ activation of cytokines, macrophages, NK cells and T cells; Initial response followed by proliferation of T cells; secondary exposure elicits more robust response than the primary response	adaptive inducible Th-1	conflicting predictions	D>S	(1, 2)
haemagglutination assay[B]	expose serially diluted plasma to dilute red blood cells from a different animal (e.g., sheep, rabbit or trout). Record agglutination on a top-lit, flat-bed scanner	measures constitutive humoral responses, including complement, NAbs/IgM. More agglutination is a stronger response.	innate constitutive Th-2	conflicting predictions	D<S	(2-5)
haemolysis/haemolytic complement assay (CH_{50}) using serum/plasma	expose serially diluted plasma to dilute red blood cells from a different animal (e.g., sheep, rabbit or trout); record lysis (happens after agglutination) on a top-lit, flat-bed scanner	measures complement-mediated innate immunity	innate constitutive	D<S	D>S	(2)

[B] Note: O'Neal and Ketterson (6) describe this as an adaptive response. This is because antibodies are produced by B cells. In some ways, this test is not that different from measuring baseline immunoglobulin levels. However, Demas (2) lists this test under hemolysis (which is innate), but says it involves natural antibodies, which are adaptive.

Table S2. Immune assays used to assess the effects of social status on immune function (page 3 of 8)

test of immune function	description of test	what the test measures	immune system components (see Table 1)	predicted direction (tradeoffs model)	predicted direction (stress-response model)	references
macrophage phagocytic ability performed on isolated cells or whole blood	isolate macrophages from blood or fluids/tissues; combine with an antigen-labeled stained yeast or SRBC; microscope is used to count the number of macrophages engulfing particles and/or the number of particles being engulfed.	measures innate immune function important for the immediate killing of numerous pathogens, from whole bacteria to virally infected and injured host cells	innate constitutive	D<S	D>S	(2, 7)
natural killer (NK) cell cytotoxicity	collect whole blood or isolate mononuclear cells from blood. Incubate with radionuclide labeled target (e.g., pathogen) cells; as target cells are destroyed by NK cells, they release Cr, which is quantified using a gamma counter	measures the cytotoxic strength of NK cell responses; NK cells are important in the initial innate response and do not require specific antigen presentation; they are important in virus resistance	innate constitutive	D<S	D>S	(2)
baseline IFN-γ levels	use ELISA to measure the concentration of IFN-γ in plasma.	measures allocation towards Th1 immunity	Th-1	D<S	D<S	(2)
baseline TNF-α levels	use ELISA to measure the concentration of TNF-α in plasma.	measures allocation towards Th1 immunity	Th-1	D<S	D<S	(2)
experimental infection with a pathogen or parasite	expose animals to a parasite/pathogen; record the success of infection (i.e., prevalence) and recovery rate and/or perform other tests of immune function	measure multiple aspects of immune function after experimental infection	integrative inducible	conflicting predictions	conflicting predictions	(2)

Table S2. Immune assays used to assess the effects of social status on immune function (page 4 of 8)

test of immune function	description of test	what the test measures	immune system components (see Table 1)	predicted direction (tradeoffs model)	predicted direction (stress-response model)	references
IFN-γ response to immune stimulants	use ELISA to measure the concentration of cytokines in plasma after blood has been exposed to an antigen	a general inflammatory response mediated by cytokines from innate and Th-1 immunity	inducible Th-1	conflicting predictions	D>S	(3)
IL-1/IL-1β response to immune stimulants	use ELISA to measure the concentration of cytokines in plasma after blood has been exposed to an antigen	high cytokine levels indicate ability to up-regulate immune signaling components, to mediate leukocyte differentiation, inflammation, and cytotoxic reactions	inducible	D>S	D>S	(3)
IL-2 response to immune stimulants	use ELISA to measure the concentration of cytokines in plasma after blood has been exposed to an antigen	high cytokine levels indicate ability to up-regulate immune signaling components, to mediate leukocyte differentiation, inflammation, and cytotoxic reactions	inducible	D>S	D>S	(3)
IL-4 response to immune stimulants	use ELISA to measure the concentration of cytokines in plasma after blood has been exposed to an antigen	inducible response mediated by Th-2 immunity	inducible Th-2	D>S	conflicting predictions	(3)
IL-6 response to immune stimulants	use ELISA to measure the concentration of cytokines in plasma after blood has been exposed to an antigen.	a general inflammatory response mediated by cytokines via Th-2 immunity	inducible Th-2	conflicting predictions	conflicting predictions	(3)

Table S2. Immune assays used to assess the effects of social status on immune function (page 5 of 8)

test of immune function	description of test	what the test measures	immune system components (see Table 1)	predicted direction (tradeoffs model)	predicted direction (stress-response model)	references
IL-10 response to immune stimulants	use ELISA to measure the concentration of cytokines in plasma after blood has been exposed to an antigen	inducible anti-inflammatory response mediated by Th-2 immunity	inducible Th-2	D>S	conflicting predictions	(3)
TNF-α response to immune stimulants	use ELISA to measure the concentration of cytokines in plasma after blood has been exposed to an antigen	a general inflammatory response mediated by cytokines from innate and th-1 immunity	inducible Th-1	conflicting predictions	D>S	(3)
sickness behaviors and/or fever in response to an antigen such as LPS	expose animal to an antigen such as LPS and measure sickness behaviors (lethargy, anorexia) and fever	measures generalized inflammatory responses to antigens; sickness responses generally include, but are not limited to fever, lethargy and mild anorexia, cytokine production and elevated glucocorticoid levels	integrative inducible	conflicting predictions	conflicting predictions	(2)
skin swelling response to injection with mitogens/antigens such as PHA, ConA, KLH and DNFB	inject skin with a novel antigen (i.e., one that the animal has NOT previously been exposed to); size of skin swelling measured with calipers.	inflammatory mediators produced by basophils; an index of basophil-mediated inflammation; perhaps some T cells, but this is less likely during the initial exposure {Martin, 2006}	inducible	D<S	conflicting predictions	(2, 3, 5)

Table S2. Immune assays used to assess the effects of social status on immune function (page 6 of 8)

test of immune function	description of test	what the test measures	immune system components (see Table 1)	predicted direction (tradeoffs model)	predicted direction (stress-response model)	references
B cell counts	count B cells via blood smears or by flow cytometry	a coarse index of constitutive, standing levels of adaptive cellular immunity; note that WBC counts can reflect current infection	not applicable	conflicting predictions	D>S	(1, 3, 8)
cell counts (cytotoxic T cells)	count cytotoxic T cells via blood smears or by flow cytometry	a coarse index of constitutive, standing levels of adaptive cellular immunity; note that WBC counts can reflect current infection	not applicable	conflicting predictions	D>S	(1, 3, 8)
cell counts (granulocytes)	count granulocytes via blood smears or by flow cytometry	a coarse index of constitutive, standing levels of cellular immunity; note that WBC counts can reflect current infection	not applicable	conflicting predictions	D>S	(1, 3, 8)
cell counts (helper T cells)	count helper T cells via blood smears or by flow cytometry	a coarse index of constitutive, standing levels of adaptive cellular immunity; note that WBC counts can reflect current infection	not applicable	conflicting predictions	D>S	(1, 3, 8)

Table S2. Immune assays used to assess the effects of social status on immune function (page 7 of 8)

test of immune function	description of test	what the test measures	immune system components (see Table 1)	predicted direction (tradeoffs model)	predicted direction (stress-response model)	references
cell counts (leukocytes)	count leukocytes via blood smears or by flow cytometry.	a coarse index of constitutive, standing levels of adaptive cellular immunity; note that WBC counts can reflect current infection	not applicable	conflicting predictions	D>S	(1, 3, 8)
cell counts (lymphocytes) [C]	Count lymphocytes via blood smears or by flow cytometry.	a coarse index of constitutive, standing levels of adaptive cellular immunity; note that WBC counts can reflect current infection	not applicable	conflicting predictions	D>S	(1, 3, 8)
cell counts (neutrophils)	Count neutrophils via blood smears or by flow cytometry.	a coarse index of constitutive, standing levels of cellular immunity; note that WBC counts can reflect current infection	not applicable	conflicting predictions	D>S	(1, 3, 8)
neutrophil (heterophil): lymphocyte ratios[D]	Create blood smears and count the number of neutrophils and lymphocytes to calculate the ratio observed in a standard number of leukocytes (e.g., 100 leukocytes).	a coarse index of energetic allocation towards innate, cell-mediated responses (neutrophils) vs. more inducible, adaptive cell-mediated responses (lymphocytes)	not applicable	D<S	D>S	(3, 9, 10)

[C]From Lee (8): Considering lymphocytes as part of constitutive immune defense is problematic; the defensive role of individual B-cells and T-cells relies upon recognition of a pathogen and a subsequent induced, adaptive response. Circulating lymphocyte concentration might be better considered a measure of investment in induced adaptive responses.

[D]The N:L ratio is a common assay of chronic stress. Stress leads to a reduction in lymphocytes and an increase in neutrophils (see (10) p. 763).

Table S2. Immune assays used to assess the effects of social status on immune function (page 8 of 8)

test of immune function	description of test	what the test measures	immune system components (see Table 1)	predicted direction (tradeoffs model)	predicted direction (stress-response model)	references
adrenal mass<br>spleen mass<br>thymus mass	measure the mass of surgically removed organs	not strongly diagnostic of immune function; heavier organs often interpreted as better immune function/higher investment in immune function, but these tissues are often enlarged during infection.	not applicable	conflicting predictions	conflicting predictions	(2, 9)
size of lymph organs (spleen, thymus, or bursa of fabricius in birds)	measure the length of surgically removed organs	not strongly diagnostic of immune function; larger organs often interpreted as better immune function/higher investment in immune function, but these tissues are often enlarged during infection	not applicable	D<S	D>S	(11)

Table S3. Tests used to assess the effects of social status on parasitism and condition (Page 1 of 1)

test of parasite burden / condition	description of test	what the test measures	references
blood parasites	use PCR or microscopy methods to quantify viral loads	higher parasite burdens may be indicative of severe infections and worse immune responses	(12)
ectoparasites	use parasitological techniques to measure the diversity of ectoparasite species or number of larvae on the skin/external body parts.	can reflect differences in exposure to parasitism as well as differences in the strength of immune responses	(13-15)
gastrointestinal parasites	Use parasitological techniques to measure the diversity of parasite species or number of eggs in fecal samples	can reflect differences in exposure to parasitism as well as differences in the strength of immune responses	(16)
hematocrit (packed cell volume) and leukocrit (height of buffy layer)	collect blood in capillary tubes and centrifuge to compact cells; hematocrit reader used to measure the packed cell volume.	a measure of physical condition; higher volumes are better; buffy coat layer might indicate WBC abundance; difficult to interpret	(1, 3, 9)

Table S4. Types of tests of immune response and sample sizes used in meta-analyses of each immune component (see Table S2 for definitions of each test).

**tests of adaptive immunity**	**sample size** (number of analyses)
baseline immunoglobulins	3
broad immunoglobulin response to challenge with an antigen	7
delayed type hypersensitivity test	7
in vitro lymphocyte proliferation to a mitogen	26
specific antibody response to challenge with an antigen	14
**tests of innate immunity**	
haemolysis/ haemolytic complement assay (ch_{50}) using serum/plasma	3
haemagglutination assay	2
macrophage phagocytic ability	3
natural killer (NK) cell cytotoxicity	9
**tests of induced immunity**	
broad immunoglobulin response to challenge with an antigen	7
cytokine response to lps or other immune stimulants	55
delayed type hypersensitivity test	7
experimental infection with a pathogen or parasite	33
in vitro lymphocyte proliferation to a mitogen	26
sickness behaviors and/or fever in response to an antigen	2
specific antibody response to challenge with an antigen	14
**tests of constitutive immunity**	
baseline immunoglobulins	3
haemagglutination assay	2
haemolysis/ haemolytic complement assay (CH_{50}) using serum/plasma	3
hematocrit (packed cell volume) and leukocrit (height of buffy layer)	3
macrophage phagocytic ability	3
natural killer (NK) cell cytotoxicity	9
**tests of Th-1-mediated immunity**	
baseline Th-1 cytokines	9
Th-1 cytokine response to LPS or other immune stimulants	13
delayed type hypersensitivity test	7
**tests of Th-2-mediated immunity**	
baseline Th-2 cytokines	5
baseline immunoglobulins	3
broad immunoglobulin response to challenge with an antigen	7
Th-2 cytokine response to LPS or other immune stimulants	24
specific antibody response to challenge with an antigen	14
haemagglutination assay	2

Table S5. List of Th-1, Th-2 and pro-inflammatory cytokines and sample sizes used in meta-analyses

**Th-1 cytokines**	**sample size** (number of analyses)
interferon-1 (IFN-1)	2
interferon-gamma (IFN-γ)	10
tumor necrosis factor-alpha (TNF-α)	10
**Th-2 cytokines**	**sample size**
interleukin-4 (IL-4)	5
interleukin-6 (IL-6)	14
interleukin-10 (IL-10)	10
**pro-inflammatory cytokines**	**sample size**
interferon-1 (IFN-1)	2
interferon-gamma (IFN-γ)	10
interleukin-6 (IL-6)	14
tumor necrosis factor-alpha (TNF-α)	10

Table S6. Measures of parasitism and sample sizes included in meta-analyses.

**parasite load**	**sample size** (number of analyses)
blood parasite	3
Ectoparasites	3
gastrointestinal parasites (including helminths and protozoans)	13
gastrointestinal parasites (helminths only)	10[a]

[a] subset of all gastrointestinal parasites

Table S7. Summary of meta-analyses for tests of immune system components for Order Rodentia, Order Primates, and Class Aves

test of immune function	sample size (analyses)	Cohen's *d*	Egger's test (p-value)	random effects model				higher in dominant or subordinate
				95% CI lower limit	95% CI upper limit	z-value	*p*-value	
tests of adaptive immunity								
Order Rodentia	43	0.037	0.051	-0.443	0.368	-0.180	0.858	neither
Order Primates	4	0.252	0.468	-0.033	0.538	1.731	0.084	neither
Class Aves	6	-0.573	0.456	-0.854	-0.291	-3.989	<.0001	dominant
tests of innate immunity								
Order Rodentia	15	-0.031	0.506	-0.865	0.803	-0.073	0.942	neither
Order Primates	0	NA	NA	NA	NA	NA	NA	NA
Class Aves	0	NA	NA	NA	NA	NA	NA	NA
tests of induced immunity								
Order Rodentia	136	0.068	0.792	-0.225	0.399	0.362	0.648	neither
Order Primates	5	0.301	0.238	-0.012	0.614	1.886	0.059	neither
Class Aves	11	-0.006	0.0016	-0.517	0.506	-0.022	0.982	neither
tests of constitutive immunity								
Order Rodentia	21	-0.025	0.771	-0.692	0.642	-0.073	0.942	neither
Order Primates	1	NA	NA	NA	NA	NA	NA	NA
Class Aves	0	NA	NA	NA	NA	NA	NA	NA
tests of Th-1 immunity								
Order Rodentia	15	0.154	0.791	-0.288	0.597	0.684	0.494	neither
Order Primates	1	NA	NA	NA	NA	NA	NA	NA
Class Aves [A]	9	-0.199	0.259	-0.719	0.320	-0.752	0.452	neither
tests of Th-2 immunity								
Order Rodentia	49	0.311	0.022	-0.096	0.718	1.496	0.135	neither
Order Primates	3	0.330	0.921	-0.056	0.716	1.676	0.094	neither
Class Aves	2	NA	NA	NA	NA	NA	NA	NA
tests of Th-1 cytokines								
Order Rodentia	15	0.154	0.791	-0.288	0.597	0.684	0.494	neither
Order Primates	1	NA	NA	NA	NA	NA	NA	NA
Class Aves	0	NA	NA	NA	NA	NA	NA	NA
tests of Th-2 cytokines								
Order Rodentia	28	0.139	0.005	-0.195	0.473	0.815	0.415	neither
Order Primates	1	NA	NA	NA	NA	NA	NA	NA
Class Aves	0	NA	NA	NA	NA	NA	NA	NA
tests of inflammatory cytokines								
Order Rodentia	35	0.255	0.015	-0.158	0.668	1.210	0.226	neither
Order Primates	1	NA	NA	NA	NA	NA	NA	NA
Class Aves	0	NA	NA	NA	NA	NA	NA	NA

[A] The moderator variable (type of immune test) significantly explains between study heterogeneity for tests of Th-1 immunity for Class Aves.

Fig. S1. *Representative journals assessing the effects of male social status on immune responses and parasitism.* The representative journals that assessed the effects of male social status on immune responses and parasitism encompassed many disciplines of biology including behavior, ecology, endocrinology, evolution, immunology, neurobiology, parasitology, and virology.

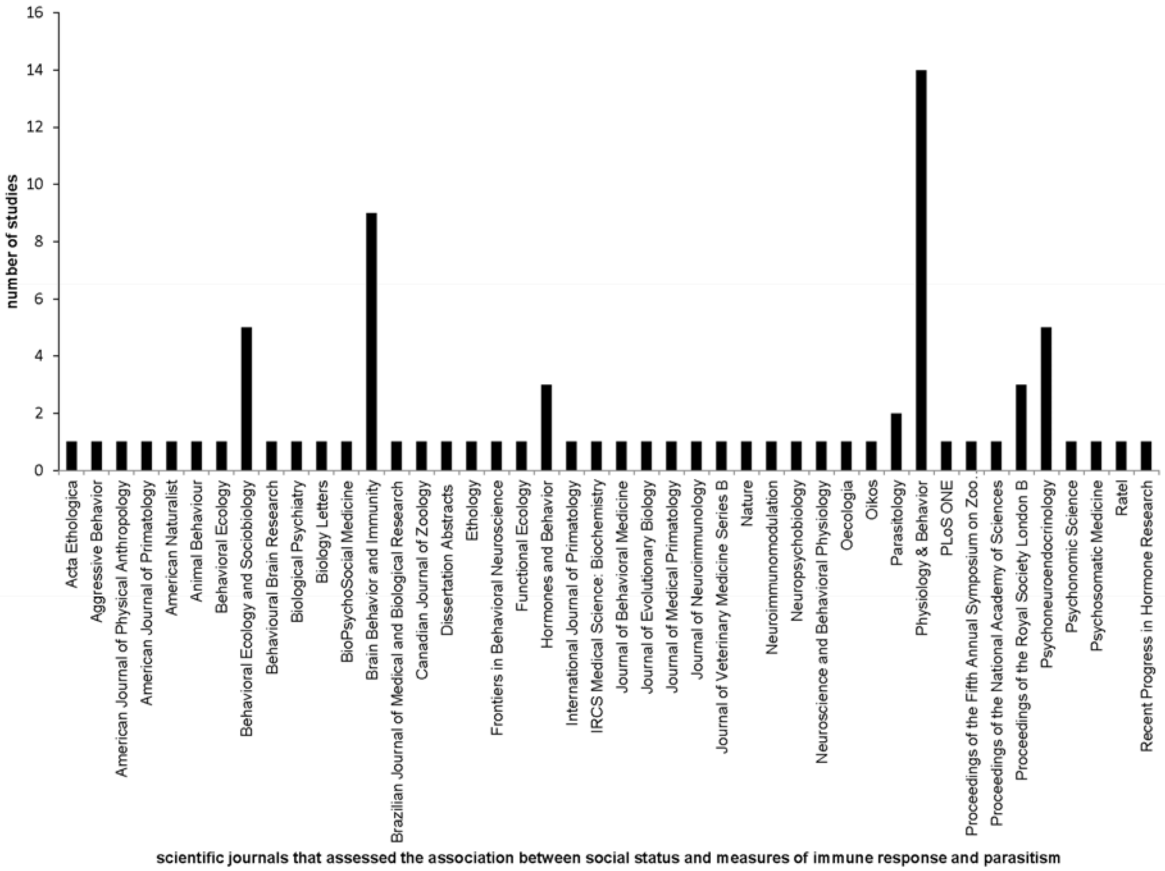

Fig. S2. *Number of representative analyses for tests of immune response.* This graph only includes tests of immune response where 3 or more analyses were available.

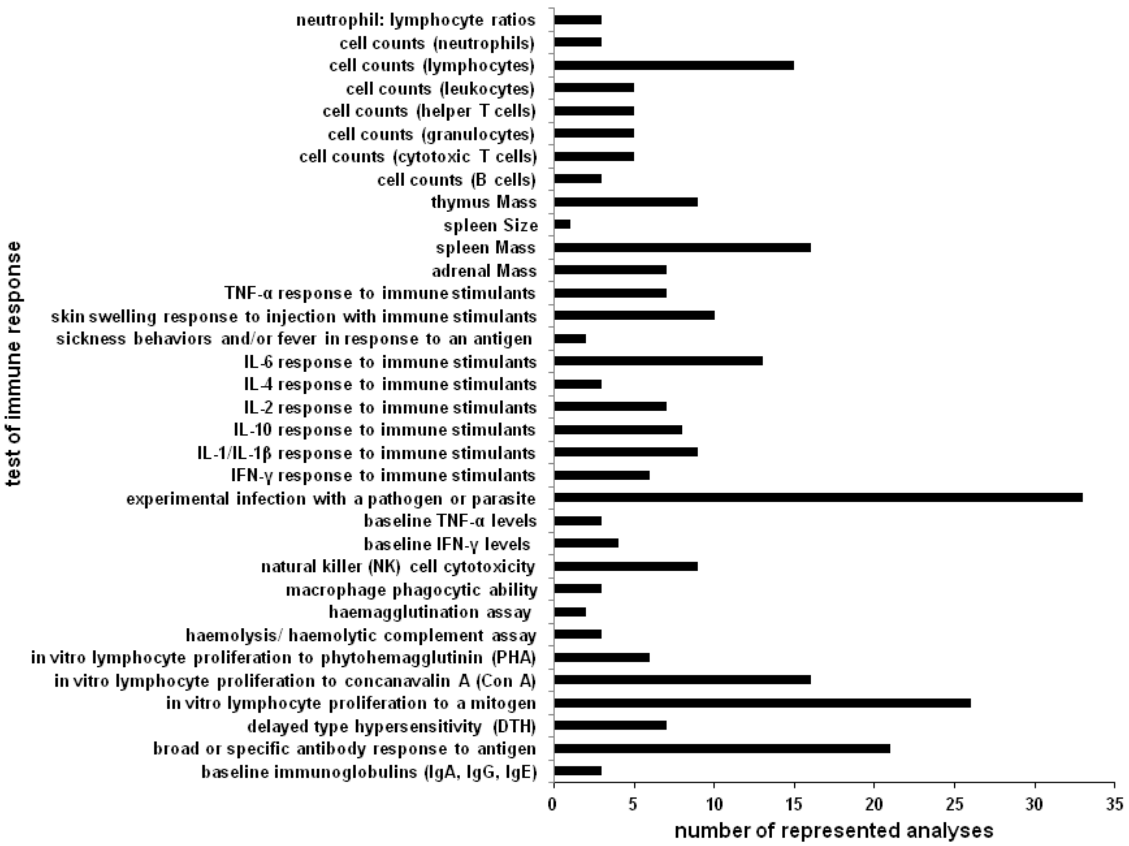

Fig. S3. *Representative taxa identified in each of the 77 studies used to assess the effects of social status on immune responses and parasitism.*

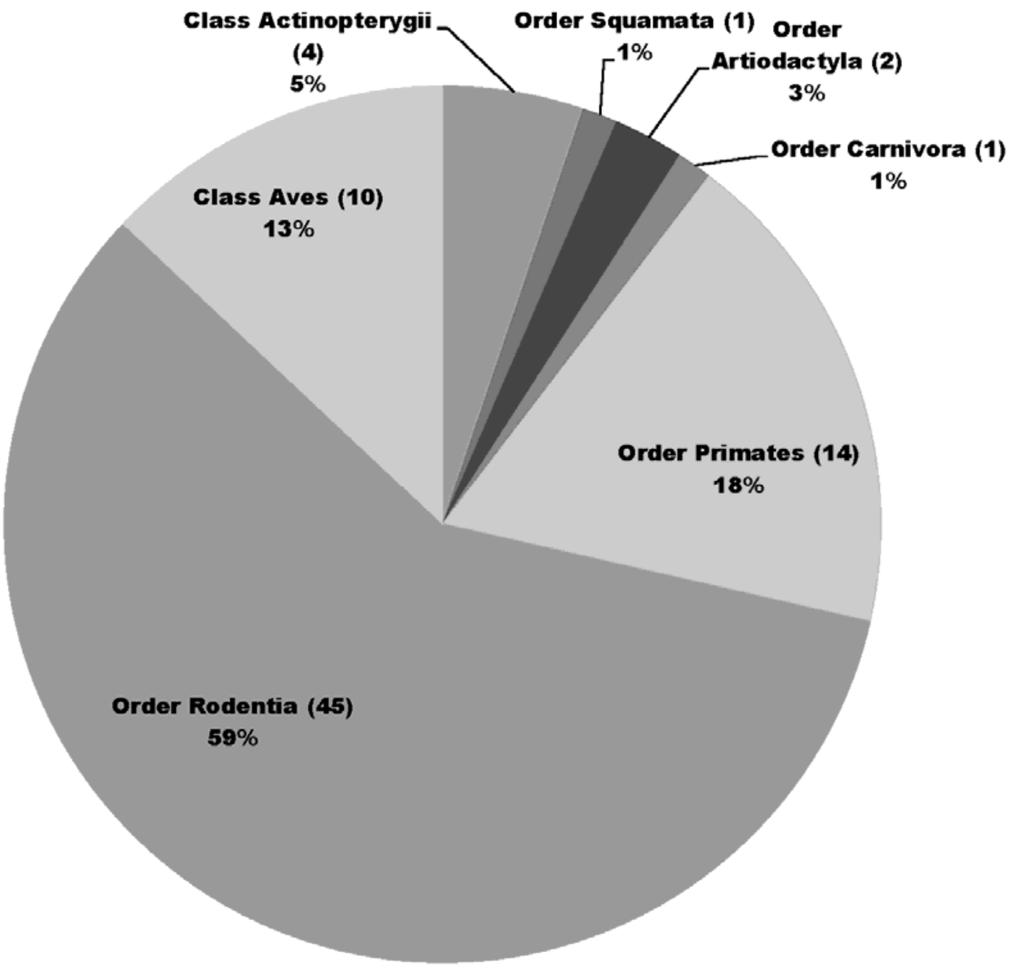

Fig. S4. *Representative mice strains that were used to assess the effects of social status on immune responses.* Note: Some studies used more than one mice strain.

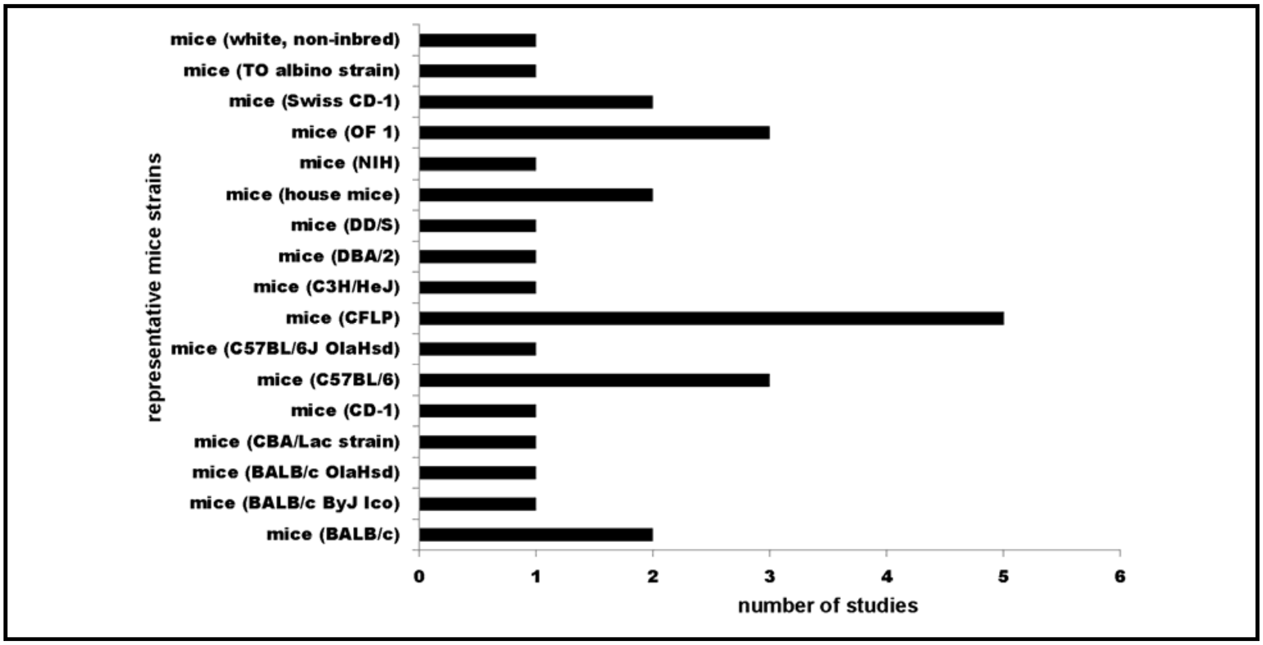

Fig. S5. *Representative rat strains that were used to assess the effects of social status on immune responses.* Note: Some studies used more than one rat strain.

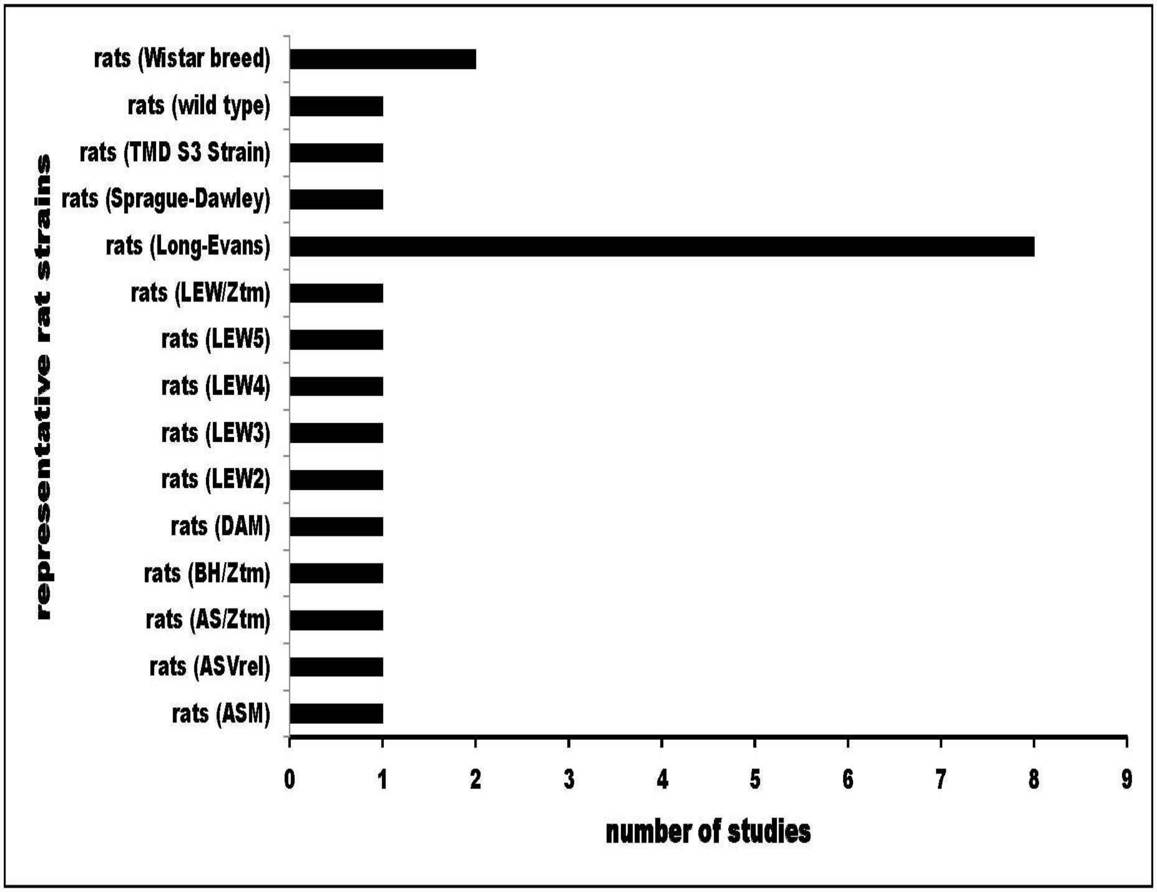

Fig. S6. *Representative primate species that were used to assess the effects of social status on immune responses and parasitism.*

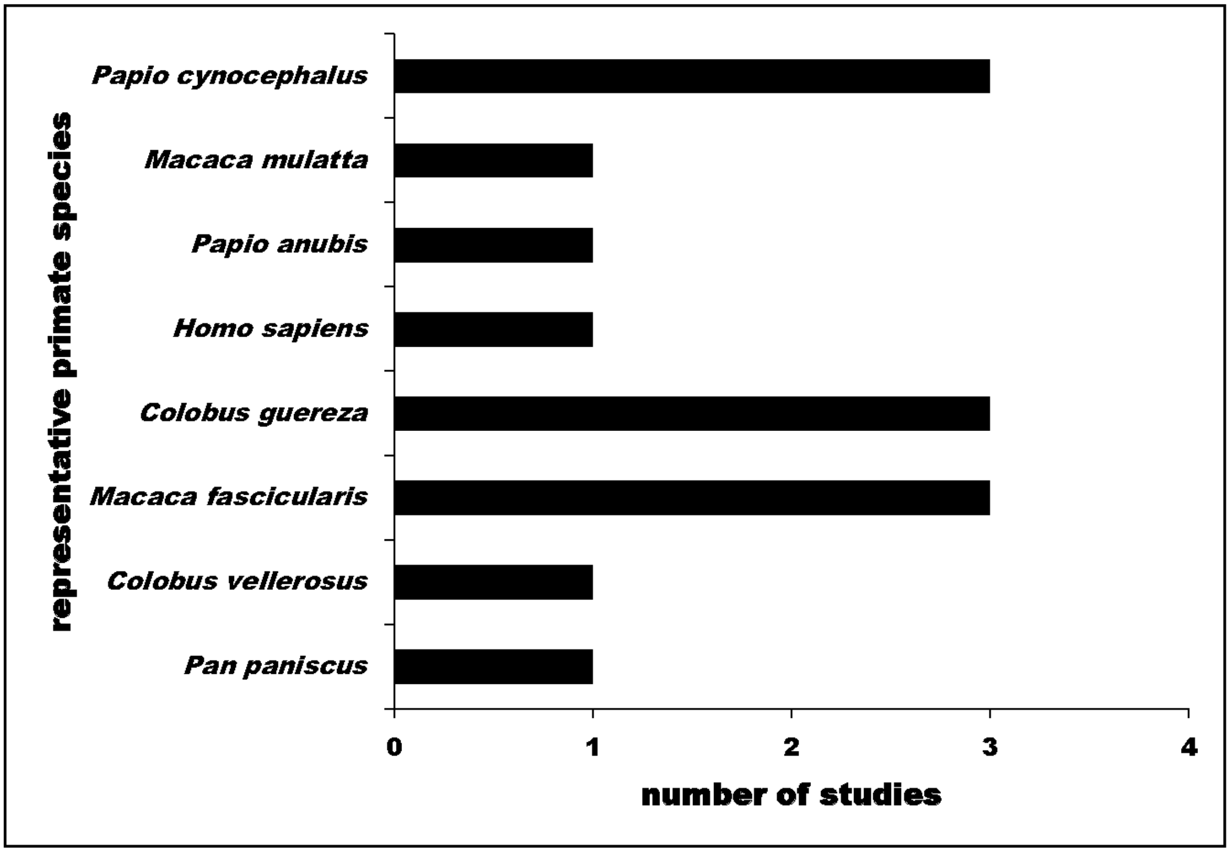

Funnel Tests (Fig. S7-S16). For each funnel plot, the horizontal axis represents the effect sizes (Cohen's *d*) for each representative study and the vertical axis represents measures of standard error for each study. Effect sizes from studies with small sample sizes tend to scatter more widely at the bottom of the plot while tests of large sample sizes tend to narrow. In the absence of publication bias, a funnel plot should represent a symmetrical, inverted funnel.

Fig. S7. *Funnel plot for tests of status-related differences in inflammatory cytokines.* An Egger's test showed significant publication bias (Egger's test: p= 0.018).

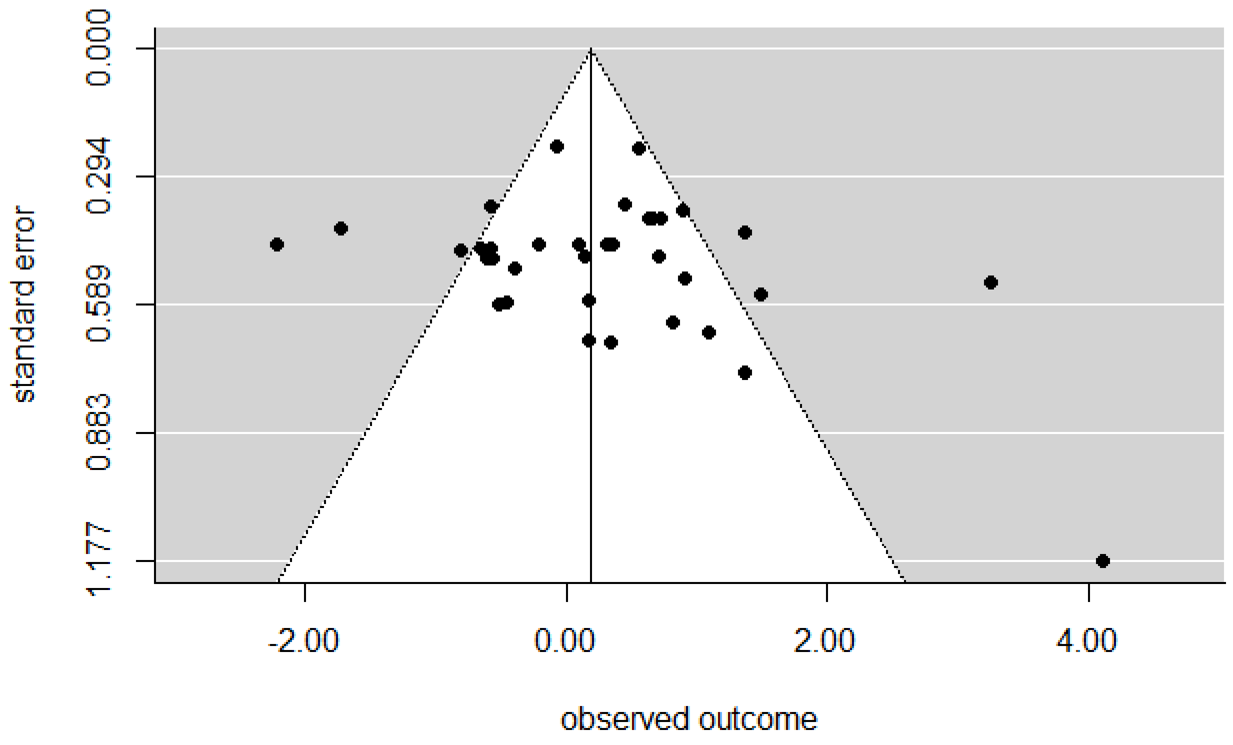

Fig. S8. *Funnel plot for tests of status-related differences in adaptive immunity.* An Egger's test showed a non-significant trend for publication bias (Egger's test: p= 0.063).

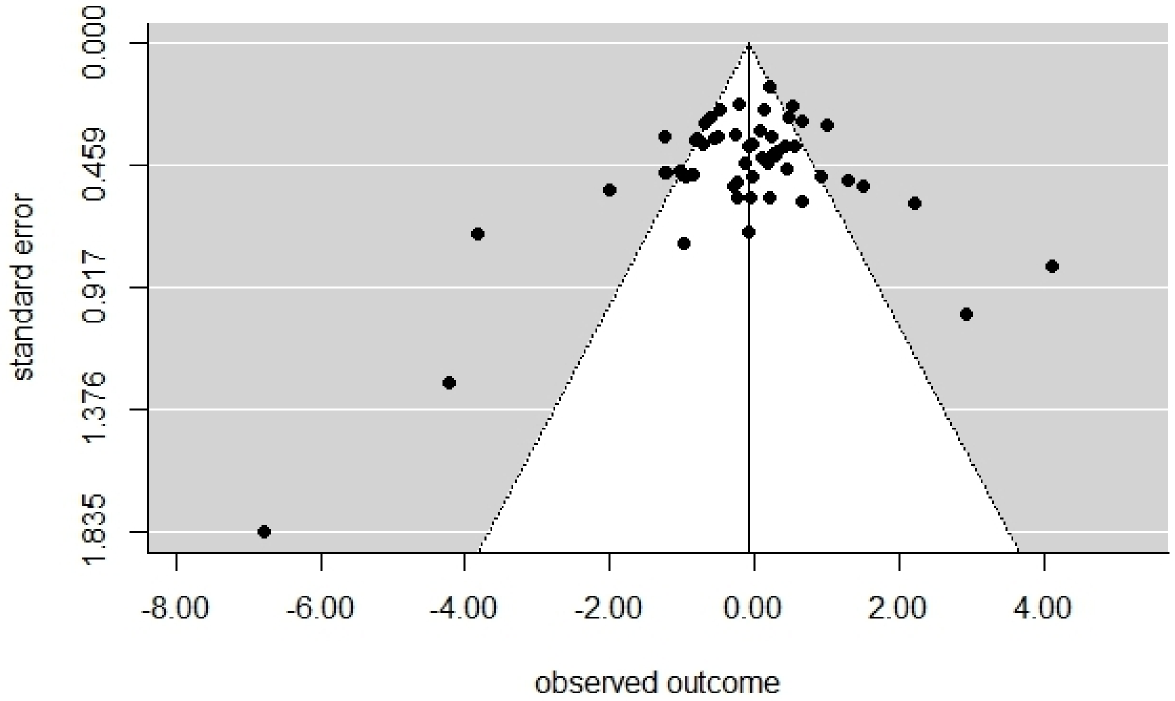

Fig. S9. *Funnel plot for tests of status-related differences in induced immunity.* An Egger's test showed no significant publication bias (Egger's test: p= 0.641).

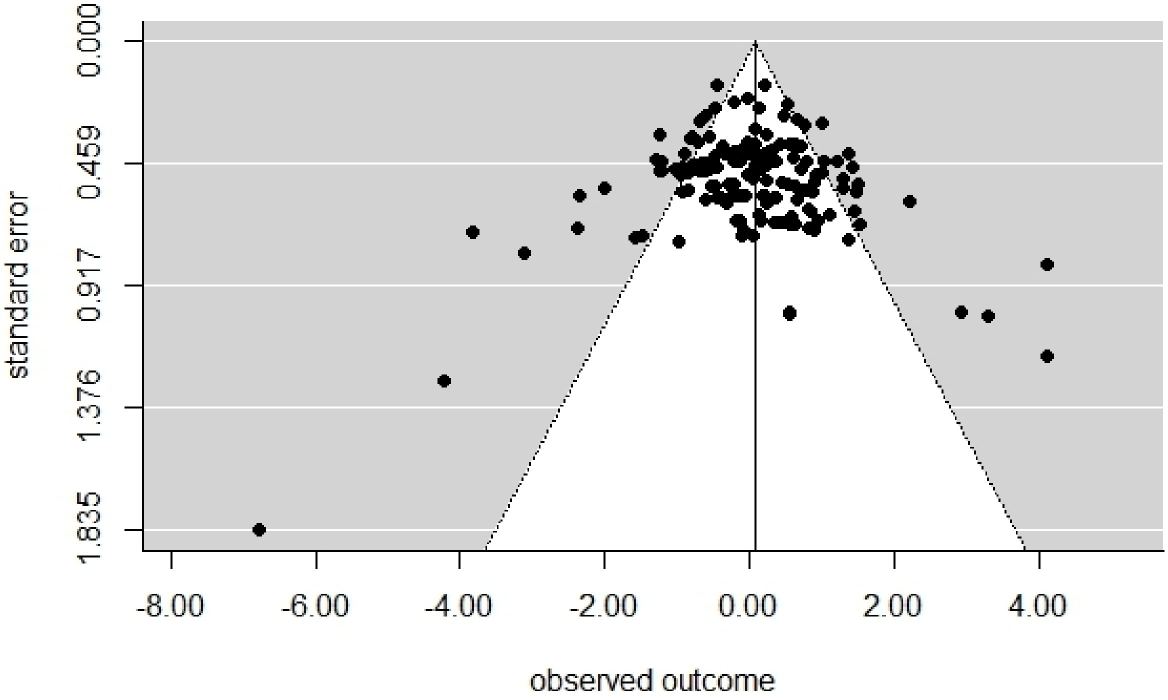

Fig. S10. *Funnel plot for tests of status-related differences in innate immunity.* An Egger's test showed no significant publication bias (Egger's test: p= 0.425).

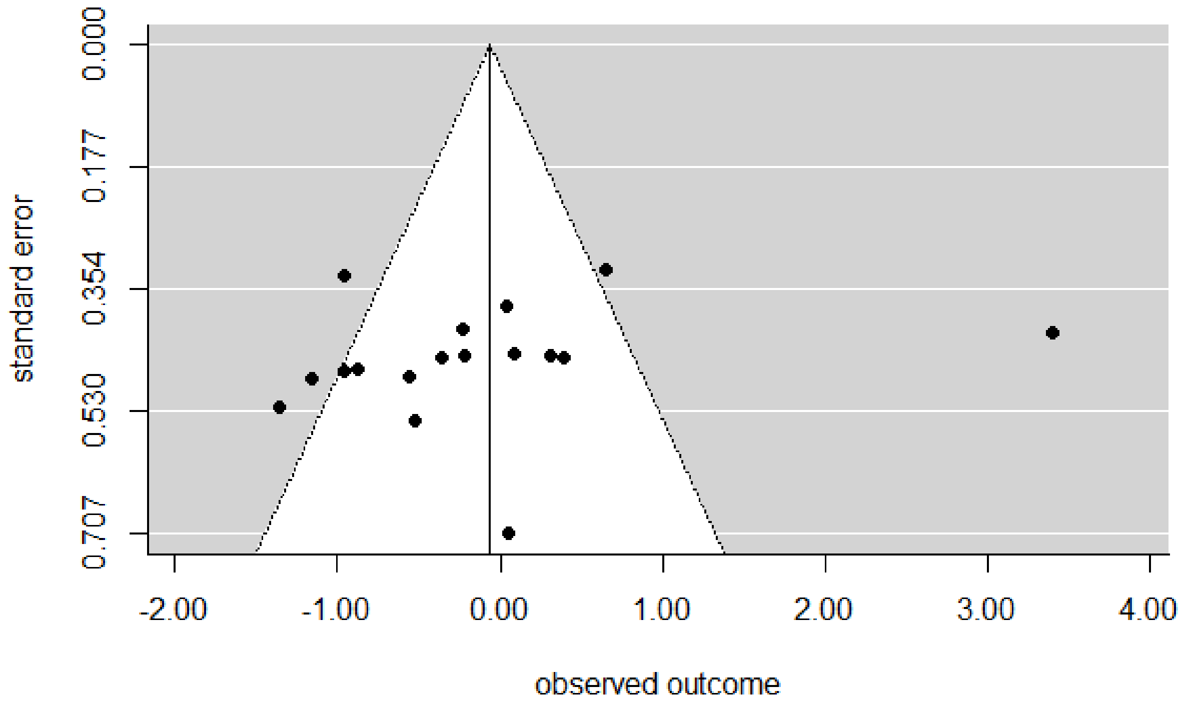

Fig. S11. *Funnel plot for tests of status-related differences in constitutive immunity*. An Egger's test showed no significant publication bias (Egger's test: p= 0.386).

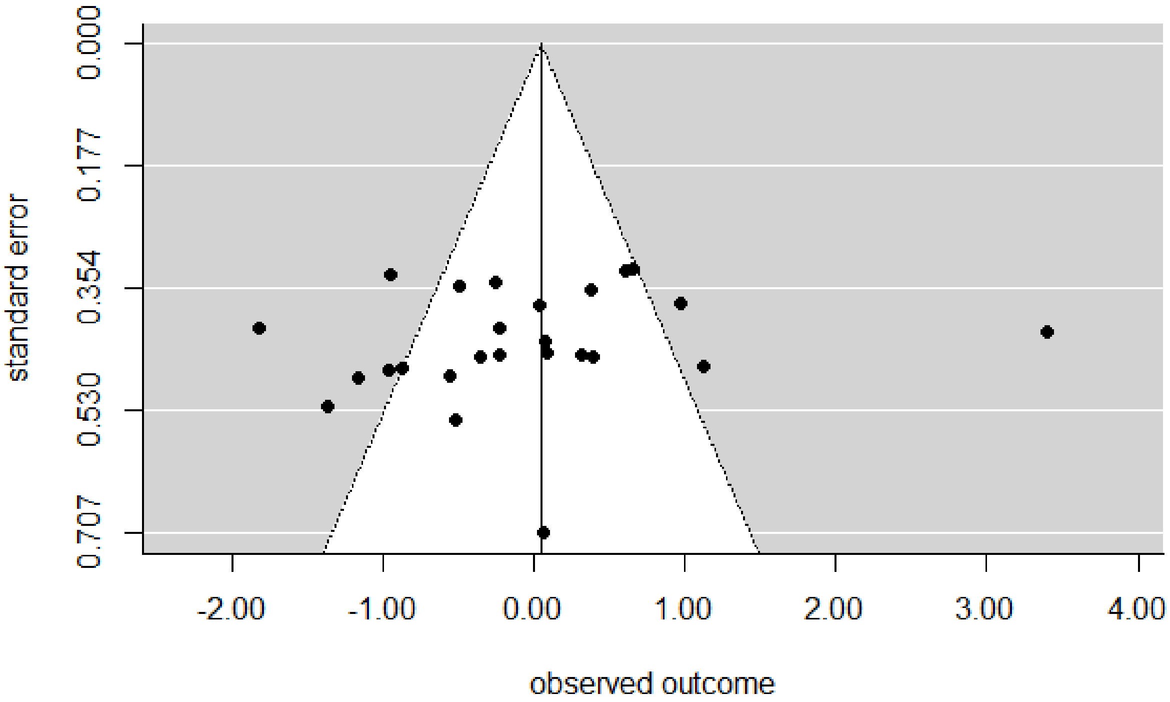

Fig. S12. *Funnel plot for tests of status-related differences in Th-1 mediated immunity.* An Egger's test showed no significant publication bias (Egger's test: p= 0.096).

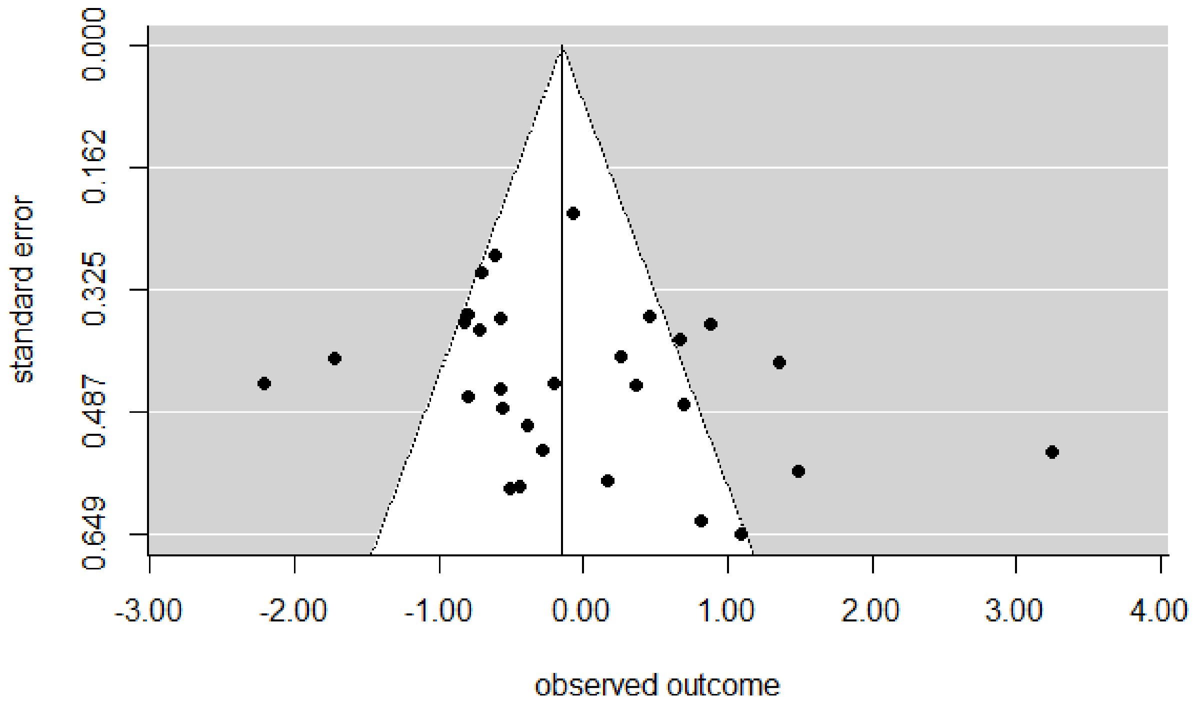

Fig. S13. *Funnel plot for tests of status-related differences in Th-2 mediated immunity immunity.* An Egger's test showed a non-significant trend for publication bias (Egger's test: p= 0.068).

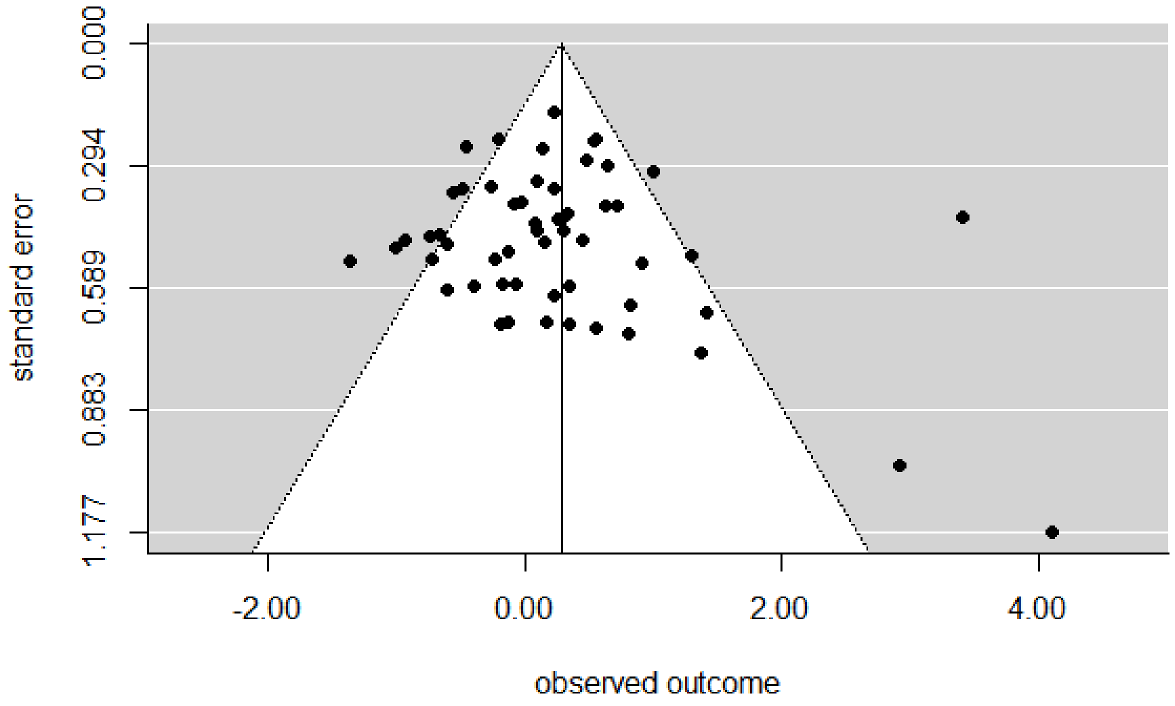

Fig. S14. *Funnel plot for tests of status-related differences in Th-1 cytokines*. An Egger's test showed no significant publication bias (Egger's test: p= 0.348).

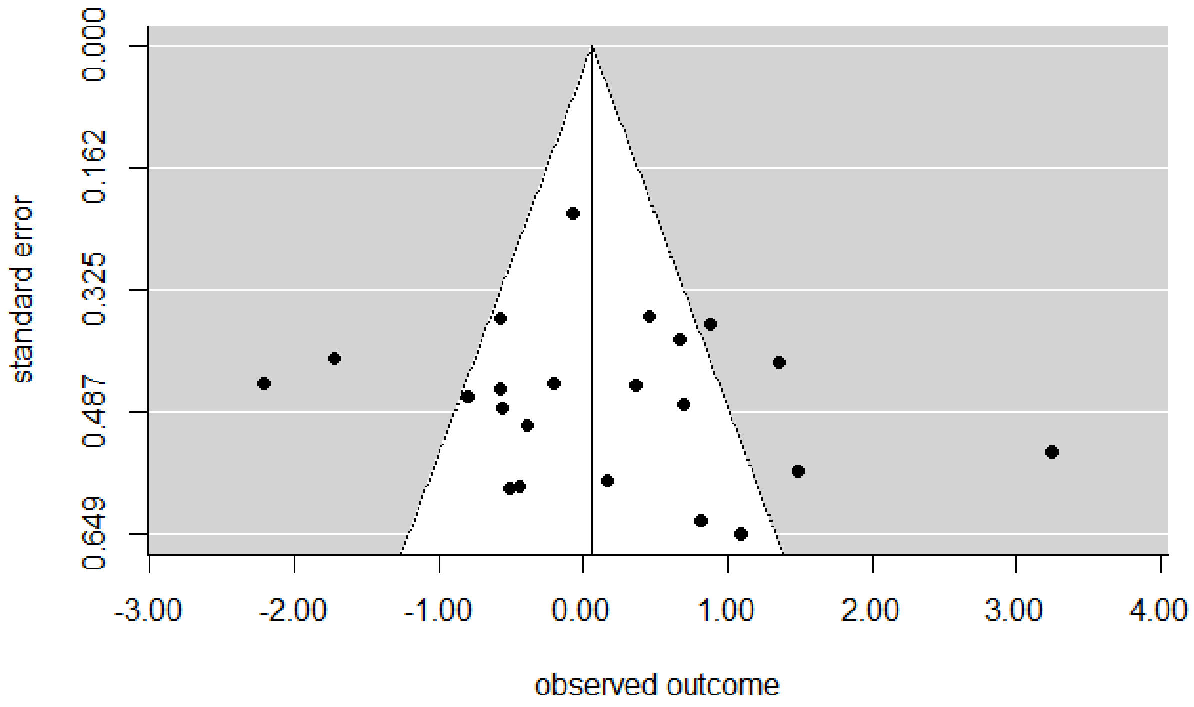

Fig. S15. *Funnel plot for tests of status-related differences in Th-2 cytokines.* An Egger's test showed a non-significant trend for publication bias (Egger's test: p= 0.063).

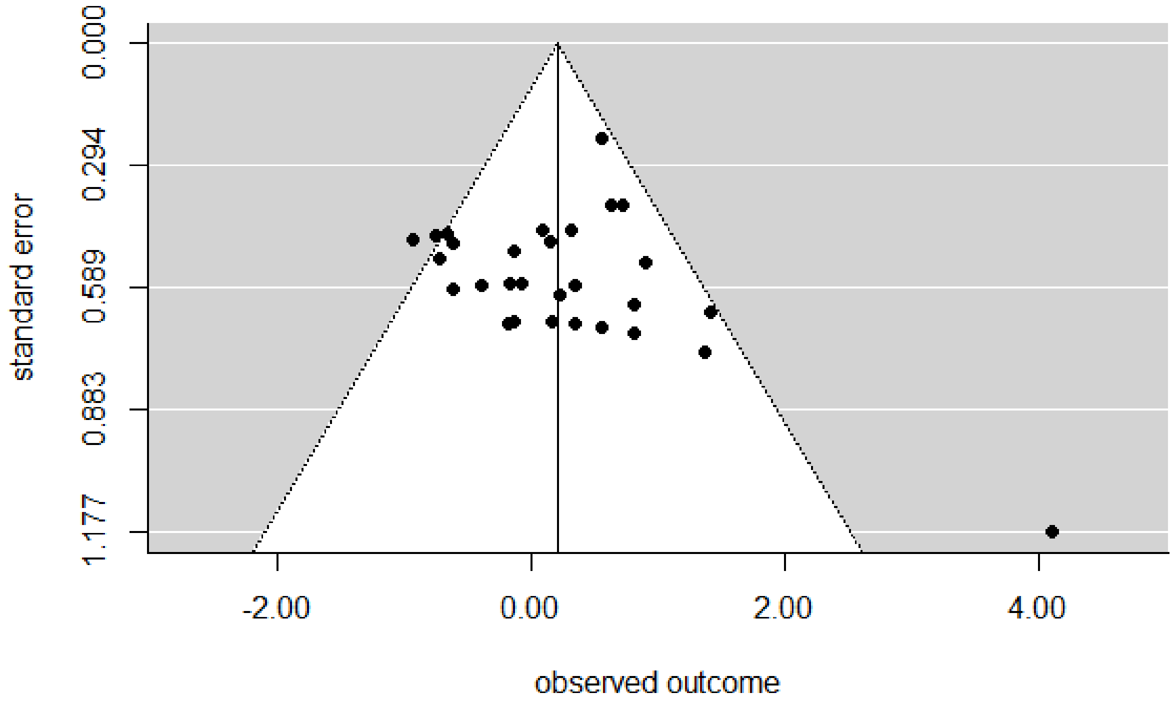

Fig. S16. *Funnel plot for tests of status-related differences in cumulative parasite burdens.* An Egger's test showed a non-significant trend for publication bias (Egger's test: p= 0.058)

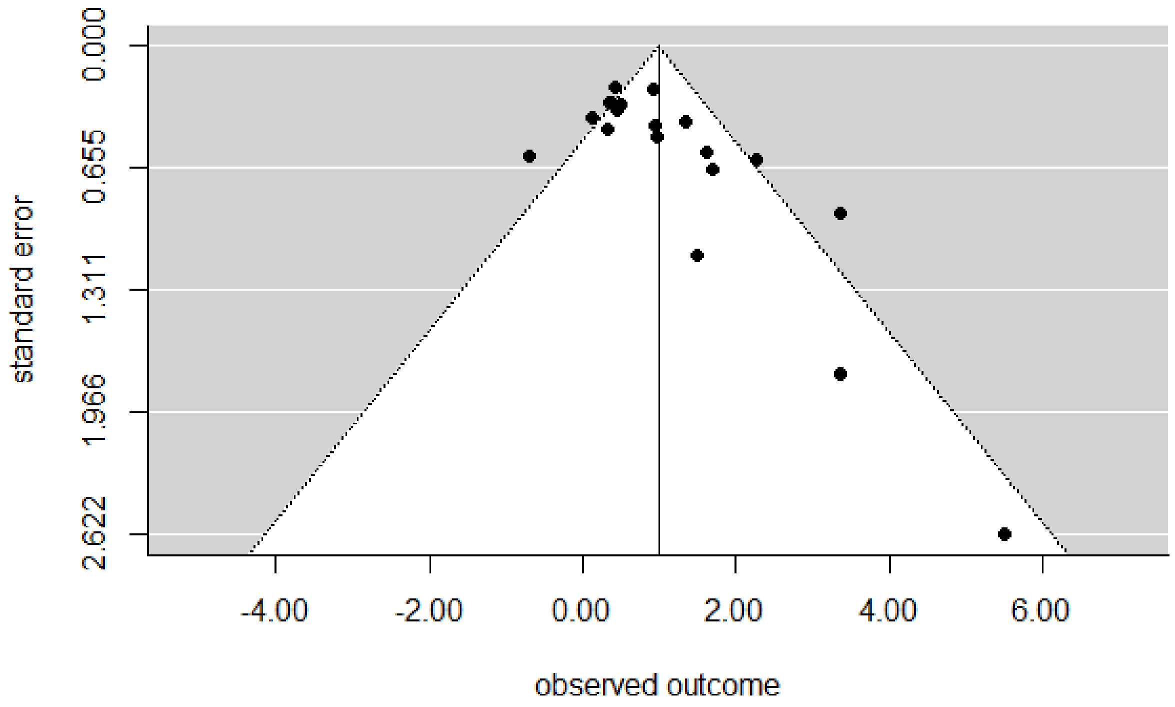

References

1. Boughton RK, Joop G, Armitage SAO. 2011 Outdoor immunology: methodological considerations for ecologists. *Funct. Ecol.* **25**, 81-100.
2. Demas GE, Zysling DA, Beechler BR, Muehlenbein MP, French SS. 2011 Beyond phytohaemagglutinin: assessing vertebrate immune function across ecological contexts. *J. Anim. Ecol.* **80**, 710-730.
3. Salvante KG. 2006 Techniques for studying integrated immune function in birds. *Auk*. **123**, 575-586.
4. Matson KD, Ricklefs RE, Klasing KC. 2005 A hemolysis-hemagglutination assay for characterizing constitutive innate humoral immunity in wild and domestic birds. *Develop. Comp. Immun.* 29, 275-286.
5. Martin LB, Han P, Lewittes J, Kuhlman JR, Klasing KC, Wikelski M. 2006 Phytohemagglutinin-induced skin swelling in birds: histological support for a classic immunoecological technique. *Funct. Ecol.* **20**, 290-299.
6. O'Neal DM, Ketterson ED. 2011 Life-history evolution, hormones, and avian immune function. In *Ecoimmunology* (eds. Demas G, Nelson R.) London: UK. Oxford University Press.
7. Millet S, Bennett J, Lee KA, Hau M, Klasing KC. 2007 Quantifying and comparing constitutive immunity across avian species. *Develop. Comp. Immunol.* **31**, 188-201.
8. Lee KA. 2006 Linking immune defenses and life history at the levels of the individual and the species. *Integr. Compar. Biol.* **46**, 1000-1015.
9. Nunn C, Altizer S. 2006 *Infectious diseases in primates: behavior, ecology, and evolution*. London: UK. Oxford University Press.
10. Davis AK, Maney DL, Maerz JC. 2008 The use of leukocyte profiles to measure stress in vertebrates: a review for ecologists. *Funct. Ecol.* **22**, 760-772.
11. Calder PC. 2007 Immunological parameters: What do they mean?'. *J. Nutr.* **137**, 773S-780S.
12. Moody AH, Chiodini PL. 2000 Methods for the detection of blood parasites. *Clin. Lab. Haematol.* **22**, 189-202.
13. Horak IG, Brown MR, Boomker J, Devos V, Vanzyl EA. 1982 Helminth and arthropod parasites of Blesbok, *Damaliscus dorcas phillipsi*, and of Bontebok, *Damaliscus dorcas dorcas*. *Onderstepoort J Vet.* **49**, 139-46.
14. Macivor KM, Horak IG, Holton KC, Petney TN. 1987 A Comparison of live and destructive sampling methods of determining the size of parasitic tick populations. *Exp. Appl. Acarol.* **3**, 131-143.
15. Vandyk PJ, McKenzie AA. 1992 An evaluation of the effectivity of the scrub technique in quantitative ectoparasite ecology. *Exp. Appl. Acarol.* **15**, 271-83.
16. Ezenwa VO. 2004 Host social behavior and parasitic infection: a multifactorial approach. *Behav.* Ecol. **15**, 446-454.
